# A Novel Cuproptosis-Related Prognostic Model and the Hub Gene *FDX1* Predict the Prognosis and Correlate with Immune Infiltration in Clear Cell Renal Cell Carcinoma

**DOI:** 10.1155/2022/2124088

**Published:** 2022-12-10

**Authors:** Kenan Zhang, Wuping Yang, Zedan Zhang, Kaifang Ma, Lei Li, Yawei Xu, Jianhui Qiu, Chaojian Yu, Jingcheng Zhou, Lin Cai, Yanqing Gong, Kan Gong

**Affiliations:** ^1^Department of Urology, Peking University First Hospital, Beijing 100034, China; ^2^Hereditary Kidney Cancer Research Center, Peking University First Hospital, Beijing 100034, China

## Abstract

Clear cell renal cell carcinoma (ccRCC) is a common malignancy of the urological system with poor prognosis. Cuproptosis is a recently discovered novel manner of cell death, and the hub gene *FDX1* could promote cuproptosis. However, the potential roles of cuproptosis-related genes (CRGs) and *FDX1* for predicting prognosis, the immune microenvironment, and therapeutic response have been poorly studied in ccRCC. In the present study, The Cancer Genome Atlas (TCGA) and Gene Expression Omnibus (GEO) data were downloaded. CRGs were subjected to prognosis analysis, and three of them were used to construct the prognostic model by least absolute shrinkage and selection operator (LASSO) regression. The CRGs prognostic model showed excellent performance. Moreover, based on the risk score of the model, the nomogram was developed to predict 1-year, 3-year, and 5-year survival. Furthermore, the hub gene of cuproptosis, *FDX1*, was an independent prognostic biomarker in multivariate Cox regression analysis. The pan-cancer analysis showed that *FDX1* was significantly downregulated and closely related to prognosis in ccRCC among 33 cancer types. Lower *FDX1* was also correlated with worse clinicopathologic features. The lower expression of *FDX1* in ccRCC was verified in the external database and our own database, which may be caused by DNA methylation. We further demonstrated that the tumor mutational burden (TMB) and immune cell infiltration were related to the expression of *FDX1*. Immune response and drug sensitivity analysis revealed that immunotherapy or elesclomol may have a favorable treatment effect in the high *FDX1* expression group and sunitinib or axitinib may work better in the low *FDX1* expression group. In conclusion, we constructed a CRGs prognostic model and revealed that *FDX1* could serve as a prognostic biomarker and predict therapeutic response in ccRCC. The study will provide a novel, precise, and individual treatment strategy for ccRCC patients.

## 1. Introduction

Renal cell carcinoma (RCC) ranks third among all genitourinary neoplasms, and most of them are clear cell renal cell carcinoma (ccRCC) [1, 2]. Partial or radical nephrectomy were the primary treatment of localized ccRCC [3]. About 30% of ccRCC patients were found in advanced stages or presented with metastases at initial diagnosis [4, 5]. In ccRCC, 70%∼90% of patients had *VHL* mutations, which were considered the first step in tumorigenesis [6]. Loss of VHL function could induce the abnormal accumulation of HIF-*α* and activation of downstream target genes, promoting the development of ccRCC [7, 8]. Nowadays, though the vast amount of data allows to enhance the reliability and accuracy of the prediction model, there were still no effective diagnostic model or markers in ccRCC [9, 10]. Moreover, while the field of molecular therapy has seen great advances, including antivascular endothelial growth factor drugs and immune inhibitors, challenges still remain for further improving prognosis because of drug resistance and adverse reactions [11, 12]. There were some emerging therapeutic strategies, such as nanomedicine, that could overcome the heterogeneity of drug response and resistance of tumors [13]. Overall, the identification of new biomarkers is important not only for predicting prognosis but also for individual therapy.

Cuproptosis is a novel manner of cell death that was recently discovered and may provide a new target for cancer treatment [14]. As we all know, there were a great number of predetermined and precisely controlled programmed cell deaths throughout the development of multicellular organisms, such as apoptosis, necroptosis, pyroptosis, and ferroptosis [15–17]. In recent decades, we have also learned that various metals could cause cell death through other pathways than apoptosis. Zinc could trigger cell death by inhibiting adenosine triphosphate (ATP) synthesis [18]. Iron could trigger ferroptosis by catalyzing the formation of toxic membrane lipid peroxides [19]. Silver-based metal ions also had cytotoxic potential in various cancer cell lines through the induction of mitochondrial damage, oxidative stress, and autophagy [20]. As for copper, the study revealed that excess copper could perturb a set of lipoylated metabolic enzymes of the tricarboxylic acid (TCA) cycle and cause the loss of iron-sulfur cluster proteins, leading to proteotoxic stress and ultimately cell death. Furthermore, the study showed a strong link between copper toxicity and mitochondrial activity. In ccRCC, HIF-1*α* could promote the shift of cellular metabolism away from the TCA cycle to glycolysis and downregulate mitochondrial respiration by regulating downstream genes such as pyruvate dehydrogenase and miR-210 [21, 22]. Therefore, we speculated that there was a tight correlation between cuproptosis-related genes and ccRCC.

In the previous study, 10 genes were found to be closely related to cuproptosis. Among them, 7 genes (*FDX1*, *LIAS*, *LIPT1*, *DLD*, *DLAT*, *PDHA1*, and *PDHB*) could promote cuproptosis, and 3 genes (*MTF1*, *GLS*, and *CDKN2A*) could inhibit cuproptosis. *FDX1*, a reductase that reduces Cu^2+^ to its more toxic form, Cu^1+^, was identified as a hub gene that regulated protein lipoylation to promote cuproptosis. Previous studies demonstrated that the FDX1 protein was not detected in the lysates of most tissues, but it was highly expressed in the adrenal gland, kidney, and testes [23]. This result may indicate the unique role of *FDX1* in kidney tissues and the development of ccRCC. The deletion of *FDX1* could cause resistance to cell death induced by one of the copper ionophores, elesclomol. In recent studies, elesclomol showed a hopeful result for the treatment of epithelial cancer, which indicated *FDX1* may serve as a biomarker candidate [24–26]. However, its role has been rarely studied, especially in ccRCC.

In this study, we downloaded and analyzed the TCGA database to construct a cuproptosis-related prognostic model in ccRCC. Moreover, we identified the hub gene *FDX1* as being downregulated, especially in KIRC, and related to DNA methylation. We also detected the association between *FDX1* and the immune microenvironment, and drug susceptibility to immunotherapy or target drugs. This study may provide an alternative model and reveal the important role of *FDX1* in predicting prognosis and therapeutic effects in ccRCC.

## 2. Materials and Methods

### 2.1. TCGA-KIRC Database and GEO Dataset Collection

**Figure 1:**
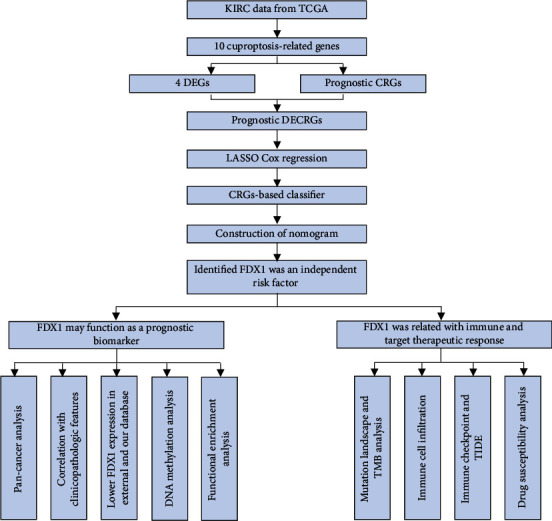
The flow sheet of this study.

The RNA-seq data, genetic mutation data, DNA methylation data, and corresponding clinical information about ccRCC tissues (*n* = 530) and adjacent normal tissues (*n* = 72) were downloaded from the TCGA-KIRC database (https://cancergenome.nih.gov/, Data Release 31, accessed on March 20, 2022). The GSE53757, GSE66271, and GSE61441 data were acquired in the Gene Expression Omnibus (GEO) databases (https://www.ncbi.nlm.nih.gov/geo, accessed on March 20, 2022) [27–29]. The single-cell databases about GSE73121 were downloaded from the CancerSEA database and analyzed (https://biocc.hrbmu.edu.cn/CancerSEA/, accessed on May 1, 2022) [30]. The single-cell data about GSE159115 was reanalyzed from the supplementary data of Zhang's study [31]. The flow sheets in this study have been shown in Figure 1.

### 2.2. Patients and Clinical Samples Collection

A total of 38 ccRCC patients enrolled in this study signed informed consent, and this research was authorized by the Ethics Committee of Peking University First Hospital (Beijing, China). All of them underwent a partial or radical nephrectomy. Fresh tumor tissues and pair-matched adjacent normal tissues were obtained from those patients. All tissue samples were immediately stored in liquid nitrogen with RNAlater solution (Thermo, AM7020, USA).

### 2.3. Differentially Expressed Genes and Functional Enrichment Analysis

The differentially expressed genes were analyzed using the “limma” R package. “Adjusted *P* < 0.05 and |Log_2_ (fold change)| ≥ 1” were defined as the threshold for the differential expression of mRNAs. Gene ontology (GO) and Kyoto Encyclopedia of Genes and Genomes (KEGG) enrichment were analyzed using the “ClusterProfiler” R package (version: 3.18.0) [32].

### 2.4. Construction and Validation of the CRGs Prognostic Model

The patients in KIRC were randomly split into the training (*n* = 352) and validation (*n* = 178) cohorts at 2 : 1 ratio, and the clinical information is summarized in Table 1. The 10 CRGs were subjected to univariate Cox regression analysis to find the genes, which could influence overall survival (OS) and progression-free survival (PFS). Then, there were three CRGs that were both significantly differentially expressed and associated with prognosis. These genes were used to construct CRGs prognostic model using least absolute shrinkage and selector operation (LASSO) analysis in the training cohort. The risk score was calculated as follows: Risk Score = *β*_1_*x*_1_ + *β*_2_*x*_2_ + *β*_3_*x*_3_+…+*β*_n_*x*_n_. The patients were divided into two different groups according to the median risk score. The prognostic model was assessed using Kaplan–Meier (KM) and time-dependent receiver operating characteristic (ROC) analysis by employing the R packages “survival” and “timeROC.” A validation cohort was used to identify the performance of this model.

### 2.5. Establishment and Evaluation of the Nomograms

We used univariate and multivariate Cox regression analyses to determine whether risk scores and other clinicopathological factors (age, gender, pathological *T* stage (pT), pathological *M* stage (pM), tumor stage, and histopathological grade) could be independent predictors of survival in ccRCC patients. Afterwards, we developed a nomogram that can assess the survival probability at 1, 3, and 5 years using all independent clinical prognostic factors. Calibration curves were drawn for 1, 3, and 5 years to compare the observed prediction probability with the actual OS probability.

### 2.6. Pan-Cancer Analysis

The expression of *FDX1* among different cancer types was detected in the GEPIA2 database (https://gepia2.cancer-pku.cn/, accessed on March 28, 2022) [33]. Univariate Cox regression analyses about OS and PFS of *FDX1* across all tumors were performed using the TCGA database. The protein level and the subcellular location of FDX1 were investigated in the Human Protein Atlas (HPA) database (https://www.proteinatlas.org/, accessed on April 16, 2022) [34, 35].

### 2.7. Mutation Gene and Copy Number Variation (CNV) Analysis

The data of mutations were visualized using the “maftools” R package [36]. Using the mutations per million bases of each sample, we calculated the tumor mutational burden (TMB) value. The KIRC CNV data of 10 CRGs was acquired and visualized from the cBioPortal database (https://www.cbioportal.org/, accessed on May 20, 2022) [37, 38].

### 2.8. Immune Cell Infiltration and Immune Checkpoint Analysis

To assess the reliability results of the immune score evaluation, we used the “immundeconv” R package and the EPIC algorithm for further analysis [39]. The algorithms had been benchmarked and had a unique advantage [40]. The expression values of *SIGLEC15*, *TIGIT*, *CD274*, *HAVCR2*, *PDCD1*, *CTLA4*, *LAG3*, and *PDCD1LG2* were determined as immune checkpoint relevant transcripts.

### 2.9. Immune Response and Drug Susceptibility Analysis

Tumor immune dysfunction and exclusion (TIDE) analysis is a computational method that integrates two mechanisms of tumor immune evasion to predict the immune checkpoint blockade (ICB) response in cancer treatment [41]. The TIDE score was calculated using TIDE online (https://tide.dfci.harvard.edu/, accessed on April 4, 2022). Predicted chemotherapeutic response for each sample based on the Genomics of Drug Sensitivity in Cancer (GDSC, https://www.cancerrxgene.org/, accessed on April 5, 2022) [42]. The prediction process was implemented by the R package “pRRophetic.” The samples' half-maximal inhibitory concentration (IC50) was estimated by ridge regression. All parameters were set to their default values. Using the batch effect of combat and tissue type of all tissues, the duplicate gene expression was summarized as a mean value.

### 2.10. Cell Culture

The 293 T human embryonic kidney cells and Caki-1, 786-O, and 769-P renal cancer cells were obtained from the National Infrastructure of Cell Line Resource (Shanghai, China). The 293 T cells were cultured in DMEM medium (Procell, PM150210, China). The Caki-1 cells were cultured in McCoy's 5A medium (Procell, PM150710, China). The 786-O and 769-P cells were cultured in RPMI 1640 medium (Procell, PM150110, China). All media contained 10% fetal bovine serum (Thermo, 10270106, USA) and penicillin-streptomycin (100 *μ*g/ml) (Gibco, 15140122, USA). All the cell cultures were maintained as a monolayer culture at 37°C in a humidified atmosphere containing 5% CO_2_.

### 2.11. RNA Extraction and RT-PCR

The total RNA of cell lines and tissues was extracted using TRIzol reagent (Sigma-Aldrich, T9424, USA) and quantified using Nanodrop 2000 (Thermo Fisher Scientific, Waltham, MA, USA). The cDNA was acquired using the TransScript First-Strand cDNA Reagent kit (TransGen, AT301, China). The ABI PRISM 7000 Fluorescent Quantitative PCR System (Thermo Fisher Scientific, USA) was used according to instruction. The reaction was performed by the following process: 95°C for 2 min, followed by 40 cycles at 95°C for 15 s and 60°C for 1 min. The primers used in this study were as follows: *FDX1*-F, TTCAACCTGTCACCTCATCTTTG,* FDX1*-R, TGCCAGATCGAGCATGTCATT; *β-actin*-F, CTGGAGAAGAGCTACGAGCTGC,* β-actin*-R, and CTAGAAGCATTTGCGGTGGACG. All the Ct values were controlled to be lower than 40 and *β-actin* was used as an internal reference. The relative expression level was calculated with the 2^−ΔΔCT^ method.

### 2.12. Western Blotting Analysis

Ice-cold radioimmunoprecipitation assay buffer (Sigma-Aldrich, R0278, USA) was used to lyse cells and total proteins were extracted. We quantified protein using BCA kit (Thermo, 23227, USA) according to the instruction. The same amount of protein was loaded for each sample and was transferred to PVDF membranes. Primary antibodies were incubated at 4°C overnight (*β*-actin, ^#^4970, Cell Signaling Technology; Anti-FDX1, ab108257, Abcam). The secondary antibody (Anti-rabbit IgG, ^#^7074, Cell Signaling Technology) was added and incubated at room temperature for 1 h. Signals were detected by chemiluminescence (ECL Western Blotting Detection Reagents, GE Healthcare) and visualized using G: BOX Chemi Gel Documentation System (Syngene, Frederick, MD, USA).

### 2.13. Statistical Analysis

Results were reported as mean ± SD for triplicate experiments unless otherwise indicated. The statistical differences of two groups were compared through the Wilcox test, and the significance difference of three groups was tested with the Kruskal–Wallis test. All analytical methods above and R packages were performed using R software version v4.0.3 (https://www.r-project.org) or GraphPad Prism v8.0.1. A value of *p* < 0.05 was considered as statistically significant, unless stated otherwise.

## 3. Results

### 3.1. The Potential Prognosis Role of 10 CRGs

**Figure 2:**
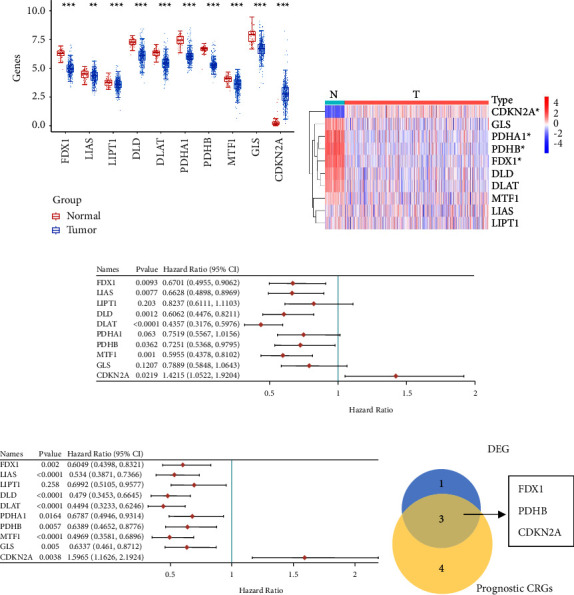
The expression and prognostic value of 10 CRGs in the TCGA database. (a) The box diagram showed the expression of 10 CRGs in KIRC. The statistical differences between normal and tumor tissues were compared through the Wilcoxon test. ^*∗*^*p* < 0.05, ^*∗∗*^*p* < 0.01, and ^*∗∗∗*^*p* < 0.001. (b) Heatmap of the expression of 10 CRGs between tumor tissues and adjacent normal tissues. Red, the level of high expression. Blue, the level of low expression. Threshold value of differentially expressed gene id adjusted *P* < 0.05 and |Log2 (fold change)| > 1. ^*∗*^*p* < 0.05. (c) The forest plot of CRGs associated with overall survival. (d) The forest plot of CRGs associated with progression-free survival. (e) Venn diagram showed the intersection of significantly low DEGs and the genes that associated with survival. DEGs, differentially expressed genes. CRGs, cuproptosis-related genes.

**Figure 3:**
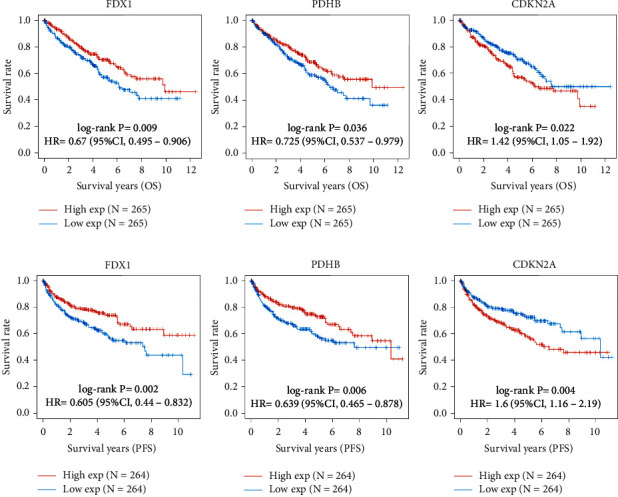
The prognostic value of three CRGs in KIRC. (a) The correlation between *FDX1*, *PDHB*, *CDKN2A*, and OS. (b) The correlation between *FDX1*, *PDHB*, *CDKN2A*, and PFS. The curve comparison with the log-rank test revealed statistically significant differences, as shown on graph. KIRC, kidney renal clear cell carcinoma. OS, overall survival. PFS, progression-free survival.

First, we investigated the expression level of 10 CRGs in ccRCC. The results showed that only *CDKN2A* was upregulated and the other 9 CRGs were downregulated in tumor tissues (Figure 2(a)). Furthermore, there were 4 significantly differentially expressed CRGs when comparing tumor tissues with adjacent normal tissues (Figure 2(b)). Univariate OS and PFS analysis showed that there were 7 CRGs that were significantly related to OS, and all 10 CRGs were significantly correlated with PFS (Figures 2(c)–2(d)). *FDX1*, *PDHB*, and *CDKN2A* were determined by taking the intersection of the significantly low DEGs and prognostic CRGs (Figure 2(e)). KM analysis showed that lower expression of *FDX1* and *PDHB* groups had a poorer OS and PFS. Higher *CDKN2A* expression group had poorer OS and PFS (Figures 3(a) and 3(b)). Interestingly, the low expression of the other 4 CRGs and 7 CRGs all showed worse OS and PFS (Supplementary Figures S1 and S2).

### 3.2. Construction and Validation of CRGs Prognostic Model

**Figure 4:**
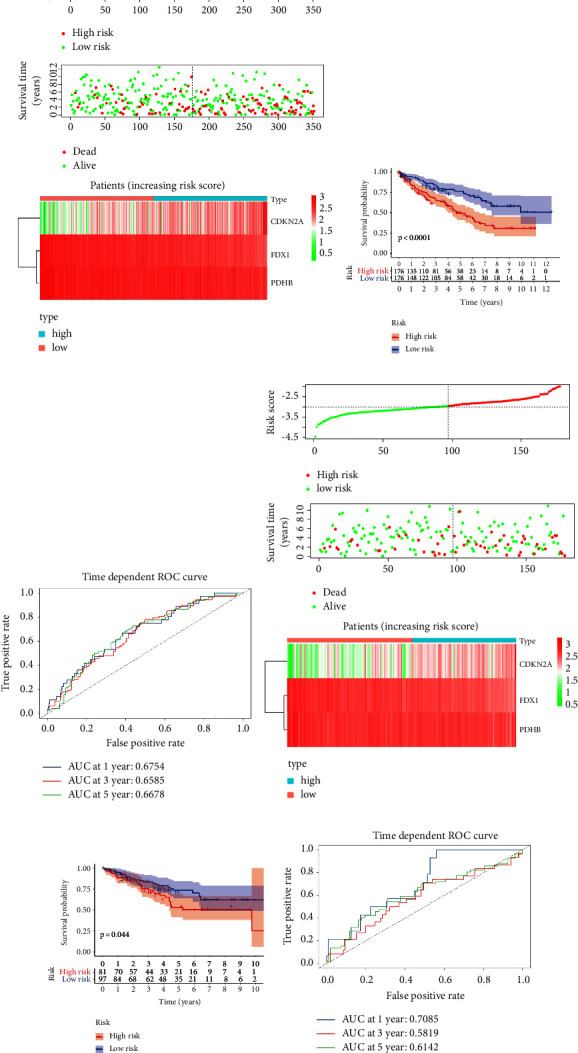
Construction and validation of the CRGs prognostic model in KIRC. (a, d) Distribution plots of the risk score, OS status, and heatmap of gene expression identified in the high-risk group compared to the low-risk group in the training cohort and validation cohort. (b, e) Kaplan–Meier analysis of OS for different risk groups in training cohort and validation cohort. The curve comparison with the log-rank test revealed statistically significant differences, as shown on graph. (c, f) ROC analysis of CRGs prognosis model in predicting the 1-, 3-, and 5-year OS in training cohort and validation cohort. CRGs, cuproptosis-related genes. OS, overall survival. ROC, receiver operating characteristic.

The LASSO Cox regression algorithm was used to construct the prognosis model in the training cohort for predicting clinical outcomes of ccRCC with CRGs. *FDX1*, *PDHB*, and *CDKN2A* were included to construct the risk model. The risk score was obtained throughout the calculation formula as follows: (−0.6044) × expression level of *FDX1* + (−0.1197) × expression level of *PDHB* + (0.2386) × expression level of *CDKN2A*. In the training cohort, based on the median cut-off value (−2.959), patients were divided into high-risk and low-risk groups (Figure 4(a)). The high-risk group had significantly worse OS based on KM analysis (Figure 4(b)). For the 1-year, 3-year, and 5-year overall survival rates, the predicted areas under the curve (AUC) were 0.675, 0.659, and 0.668, respectively (Figure 4(c)). To further validate this prognostic model, the risk score was determined in the validation cohort, and the patients were categorized into two risk groups based on the cut-off value (Figure 4(d)). Also, the high-risk group had a significant worse OS (Figure 4(e)). The predicted AUCs of the ROC curve for 1-year, 3-year, and 5-year overall survival rates were 0.709, 0.582, and 0.614 (Figure 4(f)).

### 3.3. Construction and Evaluation of Nomogram

**Figure 5:**
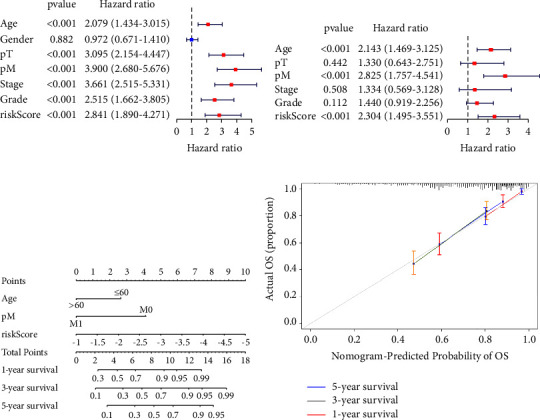
Construction and evaluation of the nomogram. (a, b). The univariate and multivariate Cox regression analyses of three CRGs combined with clinicopathological factors. (c) Score of each item of ccRCC patients were calculated according to the nomogram, and the total score after addition can predict the 1-, 3-, and 5-year survival probability. (d) The 1-, 3-, and 5-year calibration curves of the nomogram. CRGs, cuproptosis-related genes.

The age, pathologic T, pathologic M, pathologic stage, histopathological grade, and risk score could significantly affect the OS according to the univariate Cox analysis (Figure 5(a)). The multivariate Cox analysis showed that age, pathologic M, and risk score were independent risk factors for ccRCC patients in the training cohort (Figure 5(b)). Furthermore, an easy-to-use and clinically adaptable nomogram was constructed. The patients with higher total points were associated with worse 1-year, 3-year, and 5-year OS (Figure 5(c)). The calibration curve indicated the accuracy of the nomogram (Figure 5(d)).

### 3.4. Lower FDX1 Expression Showed Worse Clinicopathologic Features

**Figure 6:**
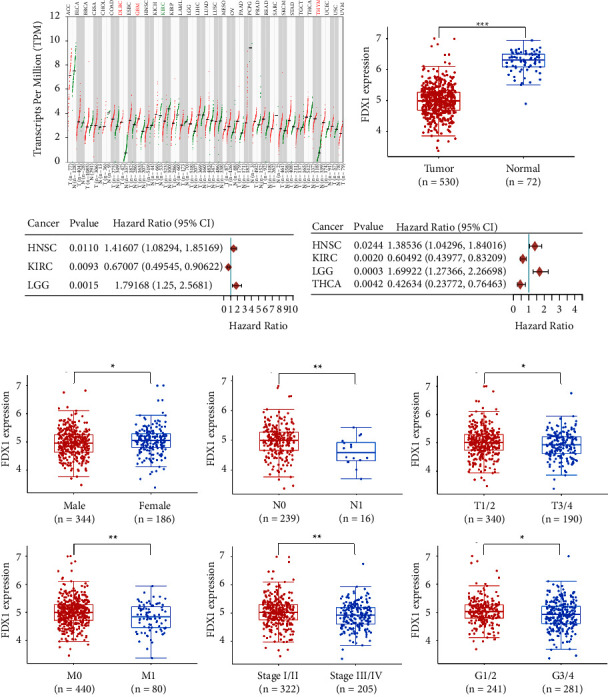
Lower expression of *FDX1* was associated with worse clinicopathologic features. (a) The expression level of *FDX1* in 33 cancer types. Green, the expression of FDX1 was significantly lower in tumor tissues. Red, the expression of *FDX1* was significantly higher in tumor tissues. The statistical differences between normal and tumor tissues were compared through the Wilcoxon test. ^*∗*^*p* < 0.05, ^*∗∗*^*p* < 0.01, and ^*∗∗∗*^*p* < 0.001. (b) The expression level of *FDX1* in tumor tissues and adjacent normal tissues in KIRC. (c) The forest plot of univariate Cox regression analysis of *FDX1* among 33 cancer types for overall survival. (d) The forest plot of univariate Cox regression analysis of *FDX1* among 33 cancer types for progression-free survival. (e) Correlations of the expression of *FDX1* and gender, T stage, N stage, M stage, tumor stage, and tumor grade. The statistical differences between different groups were compared through the Wilcoxon test. ^*∗*^*p* < 0.05, ^*∗∗*^*p* < 0.01, and ^*∗∗∗*^*p* < 0.001.

To further explore the influence of the three CRGs on ccRCC, univariate and multivariate Cox regression analyses were performed, and the results showed that only FDX1 was an independent protective factor in ccRCC (HR 0.525, 95% CI 0.338–0.816, *p* = 0.004, Table 2). Due to the critical role of *FDX1* in cuproptosis, we focused on the *FDX1* gene. The expression level of *FDX1* in different cancers was explored using the GEPIA2 database. Among the 33 cancer types in TCGA, the expression level of *FDX1* was significant lower only in KIRC when comparing tumor tissues to adjacent normal tissues (Figure 6(a) and 6(b)). Univariate Cox regression analysis found that high expression of *FDX1* had worse OS in HNSC and LGG and better OS in KIRC (Figure 6(c), Supplementary Figure S3A). Additionally, univariate Cox regression analysis also displayed that high expression of *FDX1* was associated with worse PFS in HNSC and LGG and better PFS in KIRC and THCA (Figure 6(d), Supplementary Figure S3B). The association between the expression of *FDX1* and clinicopathologic features in ccRCC was analyzed. The results showed that lower expression of *FDX1* was associated with males, a higher pathologic N stage, a higher pathologic T stage, a higher pathologic M stage, a higher pathologic stage, and a higher histologic grade (Figure 6(e), Supplementary Figures 3C and 3D).

### 3.5. Identification of the *FDX1* Expression in the External Dataset and Our Database

**Figure 7:**
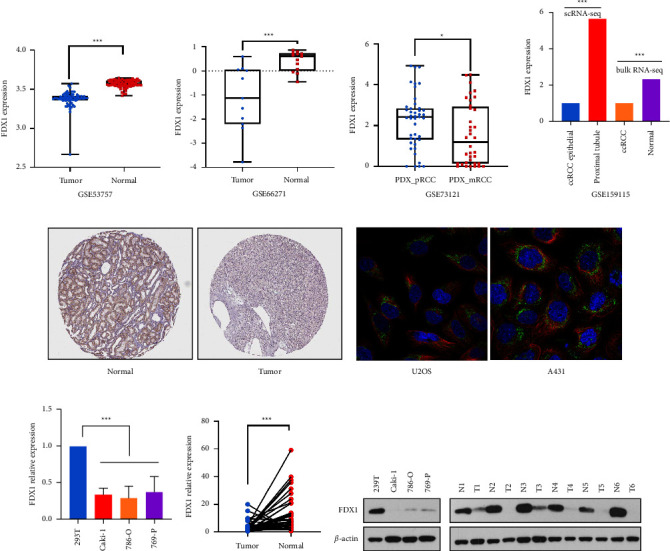
The expression level of *FDX1* in external dataset and our database. (a, b) The expression of *FDX1* was lower in tumor tissues than adjacent normal tissues based on the data from GSE53757 and GSE66271. (c) The expression of *FDX1* was lower in PDX primary tumor than PDX metastatic tumor in single cell levels based on GSE73121. (d) The expression of *FDX1* was lower in ccRCC epithelial cells than renal proximal tubule cells in single cell levels based on GSE159115. The statistical differences between different groups were compared through the Wilcoxon test. ^*∗*^*p* < 0.05, ^*∗∗*^*p* < 0.01, and ^*∗∗∗*^*p* < 0.001. (e) The protein level of FDX1 was downregulated in tumor tissues based on the HPA database. (f) The cellular distribution of FDX1 in U2OS and A431 cells. Green, FDX1 protein. Red, microtubule. Blue, nucleus. (g, h) The mRNA expression of *FDX1* in cell lines or paired tumor and adjacent normal tissues. The statistical differences between different cell lines were compared through the Kruskal–Wallis test. ^*∗*^*p* < 0.05, ^*∗∗*^*p* < 0.01, and ^*∗∗∗*^*p* < 0.001. (i) The protein expression of *FDX1* in cell lines and paired tumor and adjacent normal tissues. PDX, patient derived tumor xenograft. HPA, the Human Protein Atlas.

The expression level of *FDX1* was further identified by the GEO database in the GSE53757 and GSE66271 databases, which contained 72 and 13 paired tumor tissues and adjacent normal tissues, respectively. *FDX1* was significantly downregulated in tumor tissues (Figures 7(a) and 7(b)). We also explored the expression of *FDX1* at the single-cell RNA-seq level in the GSE73121 database, which comprised 43 PDX primary ccRCC cells and 36 PDX metastatic ccRCC cells. *FDX1* was lower expressed in metastatic ccRCC cells (Figure 7(c)). Also, we found that the single-cell RNA-seq results in the GSE159115 database demonstrated that the expression of *FDX1* was lower in ccRCC tumor epithelial cells than proximal tubule cells, and the bulk-RNA seq in this study also identified this result (Figure 7(d)). The expression of the *FDX1* protein was downregulated in tumor tissues when compared with normal tissues in the Human Protein Atlas (HPA) database (Figure 7(e)). And *FDX1* was mainly expressed in mitochondria, according to immunofluorescence results in U2OS and A431 cells from the HPA database (Figure 7(f)). To further identify the lower expression of *FDX1* in tumor tissues in our database, we performed RT-PCR in cell lines and 38 paired ccRCC tissues. The results showed *FDX1* was downregulated in Caki-1, 786-O, and 769-P cancer cell lines and 38 paired ccRCC tissues when compared with normal cells or tissues (Figures 7(g) and 7(h)). We also verified this result at the protein level, where the expression of FDX1 was downregulated in Caki-1, 786-O, and 769-P cancer cell lines and 6 paired ccRCC tissues using Western blotting analysis (Figure 7(i)).

### 3.6. The Expression of *FDX1* Was Correlated with DNA Methylation Level

**Figure 8:**
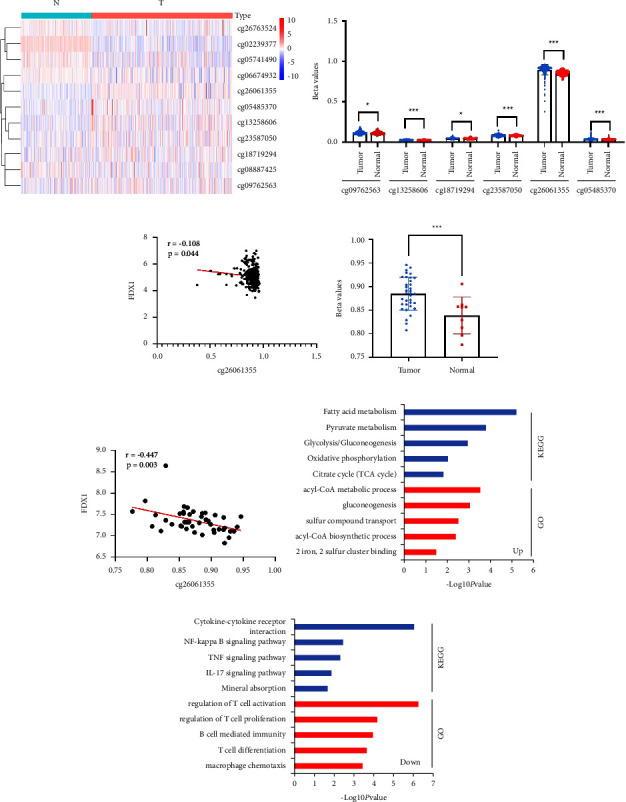
The high methylation level of *FDX1* in ccRCC. (a) Heatmap of CpG site methylation levels of 11 CpG sites in FDX1 DNA. (b) Statistical comparison of the difference in methylation levels of 6 hypermethylation CpG sites in tumor. The statistical differences between different groups were compared through the Wilcoxon test. ^*∗*^*p* < 0.05, ^*∗∗*^*p* < 0.01, and ^*∗∗∗*^*p* < 0.001. (c) Correlation between FDX1 expression and cg26061355 methylation levels in KIRC. (d) The cg26061355 methylation levels of FDX1 in GSE61441 dataset. (e) Correlation between *FDX1* expression and cg26061355 methylation levels in the GSE61441 dataset. (f, g) KEGG and GO analysis of upregulated or downregulated genes in the high *FDX1* expression group. GO, Gene Ontology; KEGG, Kyoto Encyclopedia of Genes and Genomes. Threshold value identified as significant is *p* value <0.05.

To further explore the underlying mechanism of the lower expression level of *FDX1*, we first investigated the mutation status of 10 CRGs in TCGA-KIRC. The results showed that only 2 (0.4%) of the ccRCC patients harbored copy number alterations (Supplement Figure S4A). We speculated that the low expression level of *FDX1* may relate to DNA methylation. Then, we compared the methylation levels of 11 CpG sites in *FDX1* between 323 ccRCC tissues and 160 adjacent normal tissues (Figure 8(a)). The detailed information of 11 CpG sites is shown in Table 3. The results also indicated that 6 CpG sites were hypermethylated and 5 CpG sites were hypomethylated in ccRCC tissues when compared with adjacent normal tissues (Figure 8(b) and Supplementary Figure S4B). The correlation between *FDX1* expression and the methylation level of 6 CpG sites was analyzed. Linear correlation analysis results showed that only the methylation levels of the cg26061355 site were significantly negatively correlated with *FDX1* expression (Figures 8(c)–8(e) and Supplementary Figure S4C–G).

In order to better investigate the mechanism of *FDX1* during ccRCC tumorigenesis, we compared the 75% high *FDX1* expression group with the 25% low *FDX1* expression group to find differentially expressed genes. The results showed that there were 251 genes upregulated in the high expression group and 164 genes downregulated in the high expression group (Supplementary Figure S5A and Figure S5B). GO and KEGG functional analyses were performed to explore the affected pathway that *FDX1* regulated. The results showed that fatty acid metabolism, glycolysis/gluconeogenesis, pyruvate metabolism, oxidative phosphorylation, and citrate cycle (TCA cycle) pathways were upregulated (Figure 8(f)), and cytokine-cytokine receptor interaction, NF-kappa B signaling, TNF signaling, IL-17 signaling, and mineral absorption pathways were downregulated in high expression groups (Figure 8(g), Supplementary Figure S5C–5F).

### 3.7. TMB and Immune Cell Infiltration Analysis

**Figure 9:**
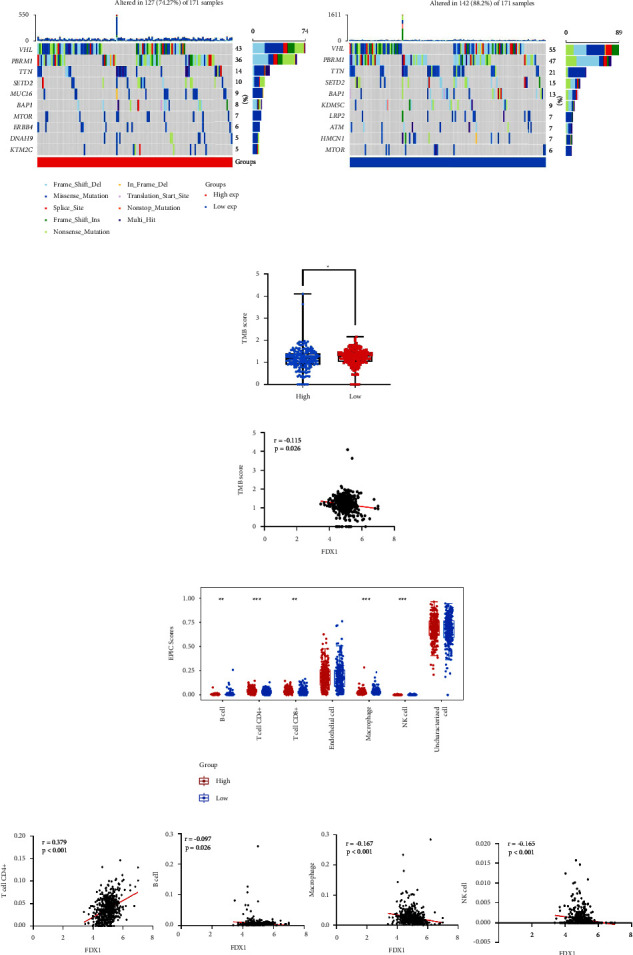
The TMB and immune cell infiltration levels in different *FDX1* expression groups in KIRC. (a) Mutation landscape of tumor in the *FDX1* high expression groups and low expression groups. (b) The score of TMB in *FDX1* high expression groups and *FDX1* low expression groups. The statistical differences between different groups were compared through the Wilcoxon test. ^*∗*^*p* < 0.05, ^*∗∗*^*p* < 0.01, ^*∗∗∗*^*p* < 0.001. (c) The correlation of TMB score and the expression of *FDX1*. (d) Comparison of the infiltration levels of T cell CD4+, T cell CD8+, endothelial cell, B cell, macrophage, and NK cell between *FDX1* high expression groups and low expression groups. The statistical differences between different groups were compared through the Wilcoxon test. ^*∗*^*p* < 0.05, ^*∗∗*^*p* < 0.01, and ^*∗∗∗*^*p* < 0.001. (e) The correlation between *FDX1* expression and infiltration levels of T cell CD4+, B cell, macrophage, and NK cell. TMB, tumor mutational burden.

The differences in mutation landscapes were found between *FDX1* high and low expression groups (Figure 9(a)). In the high *FDX1* expression group, the mutation rates were lower when compared with the low *FDX1* expression group (74.27% vs. 88.20%). Moreover, the TMB between the *FDX1* high expression group and the *FDX1* low expression group was investigated, and the result demonstrated the TMB was lower in the high expression group (Figure 9(b)). We also found that *FDX1* was downregulated in tumors with *VHL, PBRM1, SETD2, BAP1,* and *KDM5C* mutations (Supplementary Figures S6A–S6E). The TMB was negatively correlated with *FDX1* expression (Figure 9(c)). From the previous analysis of KEGG, we found that lower *FDX1* could relate to the immune microenvironment. Then, we compared immune cell infiltration between the *FDX1* high expression group and low expression group, which demonstrated that B cells, macrophages, and NK cells were higher in the *FDX1* low expression group. T cell CD4+ and T cell CD8+ were lower in the *FDX1* low expression group (Figure 9(d)). Besides, we detected a correlation between *FDX1* and different immune cells. Results revealed that the expression level of *FDX1* was positively correlated with T cell CD4+ infiltration level and negatively associated with the infiltration levels of B cells, macrophages, and NK cells (Figure 9(e), Supplementary Figures S6F–S6G).

### 3.8. The Prediction of Immune and Target Therapy Responses

**Figure 10:**
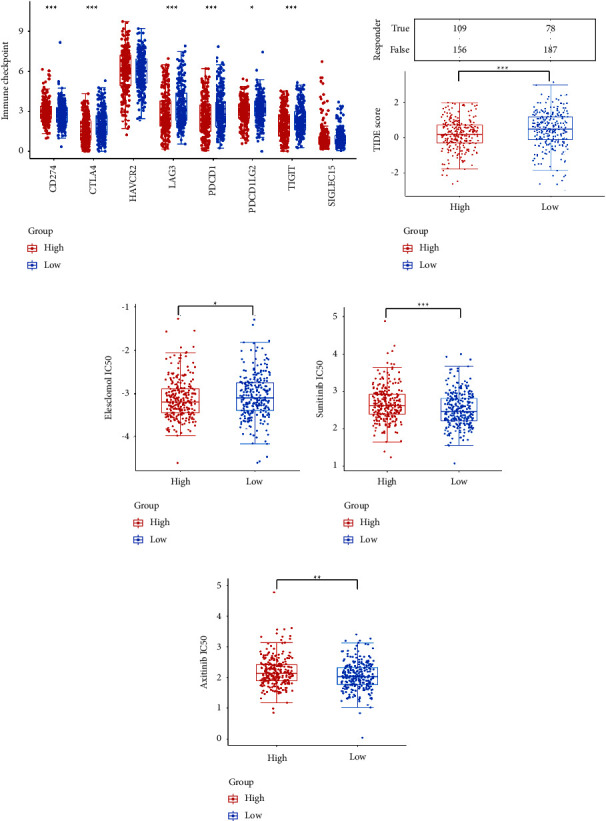
Evaluation of drug susceptibility analysis in different *FDX1* expression groups. (a) Differences in the expression levels of immune checkpoints between the high *FDX1* expression groups and low *FDX1* expression groups. (b) The distribution and statistical table of immune response in different *FDX1* expression groups in the prediction results. (c–e) Sensitivity analysis for elesclomol, sunitinib, and axitinib in *FDX1* high expression groups and low expression groups. The statistical differences between different groups were compared through the Wilcoxon test. ^*∗*^*p* < 0.05, ^*∗∗*^*p* < 0.01, and ^*∗∗∗*^*p* < 0.001.

The expression of immune checkpoint genes was detected in different *FDX1* expression groups, and the results showed that the expression levels of *CD274* were significantly higher and those of *CTLA4*, *LAG3*, *PDCD1*, *PDCD1LG1*, and *TIGIT* were significantly lower in the *FDX1* high expression group (Figure 10(a)). Moreover, the potential immune response was predicted. The results demonstrated that immune response scores were higher in the lower expression group (Figure 10(b)). Due to the fact that *FDX1* is a direct target of elesclomol, we also found that the patients in the *FDX1* high expression group were significantly more sensitive to elesclomol, and the patients in the *FDX1* low expression group were significantly more sensitive to sunitinib and axitinib (Figures 10(c)–10(e), Supplementary Figures S6H–S6J).

## 4. Disscussion

With the development of diagnostic technologies and immune checkpoint inhibitors, the survival of advanced ccRCC patients has been dramatically improved [43]. However, there were still some treatments that failed in patients with ccRCC because of metastasis, which could be attributed to the heterogeneity of ccRCC tissues [44, 45]. Cuproptosis, a novel manner of programmed cell death, is different from ferroptosis, necrosis, apoptosis, and pyroptosis. Recently, a study revealed that cuproptosis was closely associated with mitochondrial respiration. However, ccRCC is mainly dependent on the glycolytic pathway for energy because of the loss of function of VHL and the accumulation of HIF-1*α* [22, 46]. Tumor cells may develop the mechanism of cuproptosis tolerance to attenuate cell death and promote proliferation or metastasis. The role of cuproptosis in patients with ccRCC has rarely been studied. In this study, we constructed a CRGs prognosis model and further elucidated the vital role of the key gene of cuproptosis, *FDX1*, in the ccRCC. Our study about CRGs genes may shed new light on tumor classification and response to treatment.

In the present study, we screened 3 differentially expressed CRGs, *FDX1*, *PDHB*, and *CDKN2A*. All of them were associated with OS or PFS, and then they were determined to construct a CRGs prognosis model using LASSO regression analysis. The model could divide KIRC patients into high-risk and low-risk groups. The model demonstrated great survival prediction efficiency and was validated in the training and validation cohorts. Also, the ROC curve of the model in the training and validation cohorts showed moderate diagnostic performance in predicting 1-year survival (0.675 and 0.708) and 5-year survival (0.668 and 0.614). The reason that the model did not show a high level of performance may be due to the limited number of CRGs and the influence of various pathways or cell death methods. The other important pathway genes could be incorporated into this model in the future. To be more accessible for the model, we constructed a nomogram prediction model throughout, combining it with the independent clinicopathological risk factors. Then, the calibration curves showed the high accuracy of the nomogram model. In general, we constructed a novel CRGs prognosis model, which could guide clinical surveillance and treatment decisions.

To further explore the prognostic role of *FDX1*, *PDHB*, and *CDKN2A*, we performed multivariate Cox regression to identify the key gene, and the results showed that low expression of *FDX1* was an independent risk factor. Most importantly, *FDX1* was a key gene that regulated cuproptosis. *FDX1* has also been closely related to lipid-related and steroid metabolism [47, 48]. *FDX1* is essential for the synthesis of various steroid hormones (pregnenolone, aldosterone, and cortisol), and lower *FDX1* expression is associated with increased glycolysis [49, 50]. *FDX1* promoted cuproptosis through increased protein lipoylation, and the deletion of *FDX1* conferred resistance to copper-induced cell death [51]. The role of *FDX1* in cancer is rarely studied. Recently, a study showed that *FDX1* was decreased, and the patients with lower expression of *FDX1* had a worse prognosis for lung cancer [49]. The role of *FDX1* remains unclear. Therefore, we focused on the role of *FDX1* in ccRCC.

Among the expression of *FDX1* in 33 cancer types, KIRC was the only one cancer that was significantly downregulated. Lower *FDX1* expression was associated with worse OS and PFS in KIRC, which showed that *FDX1* played a vital role, especially in KIRC. The results could be attributed to the tissue specificity of *FDX1* expression. In addition, the different *FDX1* expression groups presented a significant correlation with gender, pathological T stage, lymphatic invasion, metastasis, pathological stage, and histological grade in ccRCC. On the basis of these results, we concluded that *FDX1* could be a valuable prognostic biomarker in ccRCC. Then, the lower expression of *FDX1* in tumor tissues was identified in external and our own databases. The external databases demonstrated that in the level of bulk-RNA seq or single-cell RNA seq, the *FDX1* was downregulated in tumor tissues, metastasis tumor cells and ccRCC epithelial cells. The HPA databases further confirmed this result, and the immunofluorescence analysis showed *FDX1* was mainly distributed in mitochondria. In our own database, *FDX1* was downregulated in renal cancer cell lines and paired tumor tissues at both of mRNA and protein levels. Overall, lower expression of *FDX1* was associated with worse OS and PFS, especially in ccRCC, and may function as a tumor suppressor due to being downregulated in tumor cells.

The mechanisms of low expression of *FDX1* were further explored in ccRCC. The mutation status of CRGs was detected in the cBioPortal database, and the results showed low mutation rates and copy number alternation in the *FDX1* gene. Then, we detected that the methylation level was negatively correlated with the expression of *FDX1* and verified this in the GSE61441 dataset, which indicated that DNA hypermethylation might play a vital role in decreasing the expression of *FDX1* in ccRCC. However, it was not strongly correlated between *FDX1* expression and the methylation level of cg26061355, which indicated that there may be some other mechanisms contributing to the low expression of *FDX1* in ccRCC. Previous studies reported that SF-1 and cJUN could bind with the promoter of *FDX1* in MA-10 leydig cells and ovarian granulosa cells [52, 53]. There were also some nucleotide polymorphisms in *FDX1* that may contribute to IgA nephropathy [54, 55]. All of these mechanisms may also contribute to the low expression of *FDX1* in ccRCC. Furthermore, KEGG and GO analyses were performed to explore the underlying mechanisms of *FDX1* in tumorigenesis. In the high *FDX1* expression group, oxidative phosphorylation, the citrate cycle (TCA) cycle, and the gluconeogenesis pathway were upregulated, which indicated the correlation between high *FDX1* and upregulated mitochondrial respiratory. Meanwhile, lower expression of *FDX1* may be related to the IL-17 signaling pathway and T cell activation, which indicates that low expression of *FDX1* may be related to immune cell infiltration.

The mutation landscapes were compared between two different expression groups. The TMB were higher in the low expression group. Meanwhile, TMB was negatively correlated with the expression of *FDX1*. Then, the immune cell infiltration was analyzed, and the results showed that T cell CD4+ were lower expressed and positively correlated with the low *FDX1* expression group. The B cell, macrophage, and NK cell were more highly expressed and negatively correlated with the low *FDX1* expression group. Higher B cells, macrophages, and lower T cell CD4+ were associated with poorer OS and PFS [56–59]. However, though higher NK cells were related to better OS, the population of NK cells was small in ccRCC [60]. The *CD274* was lower in the low *FDX1* expression group, as were the *CTLA4*, *LAG3*, *PDCD1*, *PDCD1LG2*, and *TIGIT*, which were higher in the low *FDX1* expression group. The results helped us better choose ICB for immune treatment or provided a theoretical basis for the development of new therapeutic strategies based on immune characteristics [61, 62]. The immune response prediction results showed that low *FDX1* expression had worse response. This may be due to low T cell CD4+ and T cell 8+ infiltration in ccRCC. This result seems to contradict the higher TMB in *FDX1* low expression groups [63, 64]. However, a recent study showed that the TMB may not have a tight correlation with ccRCC [65]. In drug sensitivity analysis, elesclomol showed a better therapeutic effect in the *FDX1* high expression group, while sunitinib and axitinib showed a worse therapeutic effect in the *FDX1* low expression group. Also, as for the *FDX1* high expression group, some new treatment strategies, such as drug delivery systems in combination with elesclomol, may have had a better performance in tumor treatment [66, 67]. Above all, these findings implied that the expression of *FDX1* could predict immune and target treatment responses in ccRCC.

Despite the strengths of our study, some limitations should be acknowledged. A larger sample size would be required for further validation of CRGs' prognostic model. In addition, more experiments are required to verify the DNA methylation and immune correlates for the hub gene of cuproptosis, *FDX1*.

On the whole, we constructed CRGs prognostic model and observed excellent performance. Then, we identified the hub gene for cuproptosis. *FDX1* was an independent prognostic biomarker, especially in ccRCC. The lower expression of *FDX1* may be related to DNA hypermethylation. *FDX1* could be a biomarker, which could predict immune and target therapeutic responses. In conclusion, our study will contribute new insights to clinical surveillance and help physicians with treatment decision-making.

## Figures and Tables

**Table 1:** Clinical features of the ccRCC patients.

Clinical feature	Training cohort	Validation cohort	Whole cohort
Overall	352	178	530
Age			
>60	176 (50.0%)	90 (50.6%)	266 (50.2%)
≤60	176 (50.0%)	88 (49.4%)	264 (49.8%)
Gender			
Male	227 (64.5)	117 (65.7%)	344 (64.9%)
Female	125 (35.5)	61 (34.3%)	186 (35.1%)
pT			
T1/T2	231 (65.6)	109 (61.2%)	340 (64.1%)
T3/T4	121 (34.4)	69 (38.8%)	190 (35.9%)
Missing			
pN			
N0	165 (46.9%)	74 (41.6%)	239 (45.1%)
N1	12 (3.4%)	4 (2.2%)	16 (3.0%)
Missing	175 (49.7%)	100 (56.2%)	275 (51.9%)
pM			
M0	292 (83.0%)	148 (83.1%)	440 (83.0%)
M1	53 (15.0%)	27 (15.2%)	80 (15.1%)
Missing	7 (2.0%)	3 (1.7%)	10 (1.9%)
Pathologic stage			
I/II	218 (61.9%)	104 (58.4%)	322 (60.8%)
III/IV	132 (37.5%)	73 (41%)	205 (38.7%)
Missing	2 (0.6%)	1 (0.6%)	3 (0.6%)
Histologic grade			
G1/G2	157 (44.6%)	84 (47.2%)	241 (45.5%)
G3/G4	189 (53.7%)	92 (51.7%)	281 (53.1%)
Missing	6 (98.3%)	2 (1.1%)	8 (1.4%)

**Table 2:** Univariate and multivariate Cox regression analyses of clinicopathologic features and the expression of *FDX1*, *PDHB*, and *CDKN2A* for OS.

Variables	Univariate analysis		Multivariate analysis	
	HR (95% CI)	*P* value	HR (95% CI)	*P* value
Age (>60 vs. ≤60)	2.079 (1.434, 3.015)	<0.001	2.150 (1.473, 3.137)	**<0.001**
Gender (male vs. female)	0.972 (0.671, 1.410)	0.882		
pT (T3/T4 vs. T1/T2)	3.095 (2.154, 4.447)	<0.001	1.388 (0.663, 2.907)	0.385
pM (M1 vs. M0)	3.900 (2.680, 5.676)	<0.001	2.836 (1.763, 4.561)	**<0.001**
Pathologic stage (III/IV vs. I/II)	3.661 (2.515, 5.331)	<0.001	1.292 (0.546, 3.059)	0.56
Histologic grade (G3/4 vs. G1/2)	2.515 (1.662, 3.805)	<0.001	1.438 (0.915, 2.262)	0.116
FDX1 (high vs. low)	0.453 (0.313, 0.656)	<0.001	0.525 (0.338, 0.816)	**0.004**
PDHB (high vs. low)	0.563 (0.369, 0.858)	0.008	1.081 (0.634, 1.845)	0.774
CDKN2A (high vs. low)	1.395 (1.161, 1.676)	<0.001	1.184 (0.973, 1.440)	0.092

pT, pathologic T stage; pM, pathologic M stage.

**Table 3:** Detailed information from 13 CpG of *FDX1* gene.

Composite element REF	Chromosome	Start	End	CGI_Coordinate	Feature_Type
cg02239377	chr11	110463373	110463374	CGI: chr11 : 110429850-110430610	—
cg05485370	chr11	110430609	110430610	CGI: chr11 : 110429850-110430610	Island
cg05741490	chr11	110429437	110429438	CGI: chr11 : 110429850-110430610	N_Shore
cg06674932	chr11	110428618	110428619	CGI: chr11 : 110429850-110430610	N_Shore
cg08887425	chr11	110429740	110429741	CGI: chr11 : 110429850-110430610	N_Shore
cg09762563	chr11	110429860	110429861	CGI: chr11 : 110429850-110430610	Island
cg13258606	chr11	110429908	110429909	CGI: chr11 : 110429850-110430610	Island
cg18719294	chr11	110429851	110429852	CGI: chr11 : 110429850-110430610	Island
cg23587050	chr11	110430404	110430405	CGI: chr11 : 110429850-110430610	Island
cg26061355	chr11	110433445	110433446	CGI: chr11 : 110429850-110430610	S_Shelf
cg26763524	chr11	110429705	110429706	CGI: chr11 : 110429850-110430610	N_Shore

## Data Availability

The data supporting the results of this study are included in the article and can be consulted with the corresponding author on reasonable request.

## Supplementary Materials

Supplementary Materials Supplementary Figure 1: The correlation between DLAT, DLD, MTF1, LIAS, and OS in KIRC. Supplementary Figure 2: The correlation between DLAT, DLD, LIPT1, MTF1, LIAS, PDHA1, GLS, and PFS in KIRC. Supplementary Figure 3: Correlation between the expression of FDX1 and clinical features. Supplementary Figure 4: The exploration of underlying mechanism of low expression of *FDX1* in tumor tissues. Supplementary Figure 5: Differential analysis between the *FDX1* high expression group and the *FDX1* low expression group. Supplementary Figure 6: Tumor mutational burden (TMB), immune infiltration, and drug susceptibility.

## References

[1] Bray F., Ferlay J., Soerjomataram I., Siegel R. L., Torre L. A., Jemal A. (2018). Global cancer statistics 2018: GLOBOCAN estimates of incidence and mortality worldwide for 36 cancers in 185 countries. *CA: A Cancer Journal for Clinicians*.

[2] Jonasch E., Walker C. L., Rathmell W. K. (2021). Clear cell renal cell carcinoma ontogeny and mechanisms of lethality. *Nature Reviews Nephrology*.

[3] Finelli A., Cheung D. C., Al-Matar A. (2020). Small renal mass surveillance: histology-specific growth rates in a biopsy-characterized cohort. *European Urology*.

[4] Makhov P., Joshi S., Ghatalia P., Kutikov A., Uzzo R. G., Kolenko V. M. (2018). Resistance to systemic therapies in clear cell renal cell carcinoma: mechanisms and management strategies. *Molecular Cancer Therapeutics*.

[5] Flippot R., Dalban C., Laguerre B. (2019). Safety and efficacy of nivolumab in brain metastases from renal cell carcinoma: results of the GETUG-AFU 26 NIVOREN multicenter phase II study. *Journal of Clinical Oncology*.

[6] van der Mijn J. C., Eng K. W., Chandra P. (2022). The genomic landscape of metastatic clear cell renal cell carcinoma after systemic therapy. *Molecular Oncology*.

[7] Huang Y., Lin D., Taniguchi C. M. (2017). Hypoxia inducible factor (HIF) in the tumor microenvironment: friend or foe?. *Science China Life Sciences*.

[8] Zhang J., Zhang Q. (2018). VHL and hypoxia signaling: beyond HIF in cancer. *Biomedicines*.

[9] Wu L., Tian X., Du H., Liu X., Wu H. (2022). Bioinformatics analysis of LGR4 in colon adenocarcinoma as potential diagnostic biomarker, therapeutic target and promoting immune cell infiltration. *Biomolecules*.

[10] Wierzbicki P. M., Klacz J., Kotulak-Chrzaszcz A. (2019). Prognostic significance of VHL, HIF1A, HIF2A, VEGFA and p53 expression in patients with clearcell renal cell carcinoma treated with sunitinib as firstline treatment. *International Journal of Oncology*.

[11] Au L., Hatipoglu E., Robert de Massy M. (2021). Determinants of anti-PD-1 response and resistance in clear cell renal cell carcinoma. *Cancer Cell*.

[12] Pal S. K., McGregor B., Suarez C. (2021). Cabozantinib in combination with atezolizumab for advanced renal cell carcinoma: results from the COSMIC-021 study. *Journal of Clinical Oncology*.

[13] Liu J., Chen C., Wei T. (2021). Dendrimeric nanosystem consistently circumvents heterogeneous drug response and resistance in pancreatic cancer. *Explorations*.

[14] Tsvetkov P., Coy S., Petrova B. (2022). Copper induces cell death by targeting lipoylated TCA cycle proteins. *Science*.

[15] Yang F., Bettadapura S. N., Smeltzer M. S., Zhu H., Wang S. (2022). Pyroptosis and pyroptosis-inducing cancer drugs. *Acta Pharmacologica Sinica*.

[16] Frank D., Vince J. E. (2019). Pyroptosis versus necroptosis: similarities, differences, and crosstalk. *Cell Death & Differentiation*.

[17] Yang W. S., Stockwell B. R. (2016). Ferroptosis: death by lipid peroxidation. *Trends in Cell Biology*.

[18] Du W., Gu M., Hu M. (2021). Lysosomal Zn(2+) release triggers rapid, mitochondria-mediated, non-apoptotic cell death in metastatic melanoma. *Cell Reports*.

[19] Zheng J., Conrad M. (2020). The metabolic underpinnings of ferroptosis. *Cell Metabolism*.

[20] Yan J., Hanif S., Zhang D. (2022). Arsenic prodrug-mediated tumor microenvironment modulation platform for synergetic glioblastoma therapy. *ACS Applied Materials & Interfaces*.

[21] Wettersten H. I., Aboud O. A., Lara P. N., Weiss R. H. (2017). Metabolic reprogramming in clear cell renal cell carcinoma. *Nature Reviews Nephrology*.

[22] Semenza G. L. (2013). HIF-1 mediates metabolic responses to intratumoral hypoxia and oncogenic mutations. *Journal of Clinical Investigation*.

[23] Sheftel A. D., Stehling O., Pierik A. J. (2010). Humans possess two mitochondrial ferredoxins, Fdx1 and Fdx2, with distinct roles in steroidogenesis, heme, and Fe/S cluster biosynthesis. *Proceedings of the National Academy of Sciences*.

[24] Monk B. J., Kauderer J. T., Moxley K. M. (2018). A phase II evaluation of elesclomol sodium and weekly paclitaxel in the treatment of recurrent or persistent platinum-resistant ovarian, fallopian tube or primary peritoneal cancer: an NRG oncology/gynecologic oncology group study. *Gynecologic Oncology*.

[25] O’Day S. J., Eggermont A. M., Chiarion-Sileni V. (2013). Final results of phase III SYMMETRY study: randomized, double-blind trial of elesclomol plus paclitaxel versus paclitaxel alone as treatment for chemotherapy-naive patients with advanced melanoma. *Journal of Clinical Oncology*.

[26] Hoefflin R., Harlander S., Schäfer S. (2020). HIF-1*α* and HIF-2*α* differently regulate tumour development and inflammation of clear cell renal cell carcinoma in mice. *Nature Communications*.

[27] von Roemeling C. A., Radisky D. C., Marlow L. A. (2014). Neuronal pentraxin 2 supports clear cell renal cell carcinoma by activating the AMPA-selective glutamate receptor-4. *Cancer Research*.

[28] Wotschofsky Z., Gummlich L., Liep J. (2016). Integrated microRNA and mRNA signature associated with the transition from the locally confined to the metastasized clear cell renal cell carcinoma exemplified by miR-146-5p. *PLoS One*.

[29] Wei J. H., Haddad A., Wu K. J. (2015). A CpG-methylation-based assay to predict survival in clear cell renal cell carcinoma. *Nature Communications*.

[30] Yuan H., Yan M., Zhang G. (2019). CancerSEA: a cancer single-cell state atlas. *Nucleic Acids Research*.

[31] Zhang Y., Narayanan S. P., Mannan R. (2021). Single-cell analyses of renal cell cancers reveal insights into tumor microenvironment, cell of origin, and therapy response. *Proceedings of the National Academy of Sciences of the USA*.

[32] Yu G., Wang L. G., Han Y., He Q. Y. (2012). clusterProfiler: an R package for comparing biological themes among gene clusters. *OMICS: A Journal of Integrative Biology*.

[33] Tang Z., Li C., Kang B., Gao G., Li C., Zhang Z. (2017). GEPIA: a web server for cancer and normal gene expression profiling and interactive analyses. *Nucleic Acids Research*.

[34] Uhlen M., Fagerberg L., Hallstrom B. M. (2015). Proteomics. Tissue-based map of the human proteome. *Science*.

[35] Thul P. J., Akesson L., Wiking M. (2017). A subcellular map of the human proteome. *Science*.

[36] Mayakonda A., Lin D. C., Assenov Y., Plass C., Koeffler H. P. (2018). Maftools: efficient and comprehensive analysis of somatic variants in cancer. *Genome Research*.

[37] Gao J., Aksoy B. A., Dogrusoz U. (2013). Integrative analysis of complex cancer genomics and clinical profiles using the cBioPortal. *Science Signaling*.

[38] Cerami E., Gao J., Dogrusoz U. (2012). The cBio cancer genomics portal: an open platform for exploring multidimensional cancer genomics data. *Cancer Discovery*.

[39] Sturm G., Finotello F., Petitprez F. (2019). Comprehensive evaluation of transcriptome-basedcell-type quantification methods for immuno-oncology. *Bioinformatics*.

[40] Racle J., Gfeller D. (2020). EPIC: a tool to estimate the proportions of different cell types from bulk gene expression data. *Methods in Molecular Biology*.

[41] Jiang P., Gu S., Pan D. (2018). Signatures of T cell dysfunction and exclusion predict cancer immunotherapy response. *Nature Medicine*.

[42] Yang W., Soares J., Greninger P. (2012). Genomics of Drug Sensitivity in Cancer (GDSC): a resource for therapeutic biomarker discovery in cancer cells. *Nucleic Acids Research*.

[43] Barata P. C., Rini B. I. (2017). Treatment of renal cell carcinoma: current status and future directions. *CA: A Cancer Journal for Clinicians*.

[44] Lopez J. I., Angulo J. C. (2018). Pathological bases and clinical impact of intratumor heterogeneity in clear cell renal cell carcinoma. *Current Urology Reports*.

[45] Dabestani S., Thorstenson A., Lindblad P., Harmenberg U., Ljungberg B., Lundstam S. (2016). Renal cell carcinoma recurrences and metastases in primary non-metastatic patients: a population-based study. *World Journal of Urology*.

[46] Clark D. J., Dhanasekaran S. M., Petralia F. (2019). Integrated proteogenomic characterization of clear cell renal cell carcinoma. *Cell*.

[47] Peden E. A., Boehm M., Mulder D. W. (2013). Identification of global ferredoxin interaction networks in Chlamydomonas reinhardtii. *Journal of Biological Chemistry*.

[48] Wang Z., Dong H., Yang L., Yi P., Wang Q., Huang D. (2021). The role of FDX1 in granulosa cell of Polycystic ovary syndrome (PCOS). *BMC Endocrine Disorders*.

[49] Zhang Z., Ma Y., Guo X. (2021). FDX1 can impact the prognosis and mediate the metabolism of lung adenocarcinoma. *Frontiers in Pharmacology*.

[50] Cormier M., Ghouili F., Roumaud P., Martin L. J., Touaibia M. (2017). Influence of flavonols and quercetin derivative compounds on MA-10 Leydig cells steroidogenic genes expressions. *Toxicology in Vitro*.

[51] Tsvetkov P., Detappe A., Cai K. (2019). Mitochondrial metabolism promotes adaptation to proteotoxic stress. *Nature Chemical Biology*.

[52] Imamichi Y., Mizutani T., Ju Y. (2013). Transcriptional regulation of human ferredoxin 1 in ovarian granulosa cells. *Molecular and Cellular Endocrinology*.

[53] Roumaud P., Rwigemera A., Martin L. J. (2017). Transcription factors SF1 and cJUN cooperate to activate the Fdx1 promoter in MA-10 Leydig cells. *The Journal of Steroid Biochemistry and Molecular Biology*.

[54] Niu D., Gao Y., Xie L. (2015). Genetic polymorphisms in TNFSF13 and FDX1 are associated with IgA nephropathy in the Han Chinese population. *Human Immunology*.

[55] Niu D., Ren Y., Xie L. (2015). Association between CCDC132, FDX1 and TNFSF13 gene polymorphisms and the risk of IgA nephropathy. *Nephrology*.

[56] Roumenina L. T., Daugan M. V., Noe R. (2019). Tumor cells hijack macrophage-produced complement C1q to promote tumor growth. *Cancer Immunology Research*.

[57] Sjoberg E., Frodin M., Lovrot J. (2018). A minority-group of renal cell cancer patients with high infiltration of CD20+B-cells is associated with poor prognosis. *British Journal of Cancer*.

[58] Diaz-Montero C. M., Rini B. I., Finke J. H. (2020). The immunology of renal cell carcinoma. *Nature Reviews Nephrology*.

[59] Komohara Y., Hasita H., Ohnishi K. (2011). Macrophage infiltration and its prognostic relevance in clear cell renal cell carcinoma. *Cancer Science*.

[60] Eckl J., Buchner A., Prinz P. U. (2012). Transcript signature predicts tissue NK cell content and defines renal cell carcinoma subgroups independent of TNM staging. *Journal of Molecular Medicine (Berlin, Germany)*.

[61] Ziqi W., Kai C., Costabel U., Xiaoju Z. (2022). Nanotechnology-facilitated Vaccine Development during the Coronavirus Disease 2019 (COVID-19) Pandemic. *Exploration*.

[62] Qiao N., Du G., Zhong X., Sun X. (2021). Recombinant lactic acid bacteria as promising vectors for mucosal vaccination. *Explorations*.

[63] Rizvi N. A., Hellmann M. D., Snyder A. (2015). Mutational landscape determines sensitivity to PD-1 blockade in non–small cell lung cancer. *Science*.

[64] Alexandrov L. B., Nik-Zainal S., Wedge D. C. (2013). Signatures of mutational processes in human cancer. *Nature*.

[65] McDermott D. F., Huseni M. A., Atkins M. B. (2018). Clinical activity and molecular correlates of response to atezolizumab alone or in combination with bevacizumab versus sunitinib in renal cell carcinoma. *Nature Medicine*.

[66] Wu H., Chen Q., Jiao M. (2020). Evaluation of nanomechanical properties of hyperbranched polyglycerols as prospective cell membrane engineering block. *Colloids and Surfaces B Biointerfaces*.

[67] Guo S., Li K., Hu B. (2021). Membrane-destabilizing ionizable lipid empowered imaging-guided siRNA delivery and cancer treatment. *Explorations*.

