# Supplementary material for: A Novel Cuproptosis-Related Prognostic Model and the Hub Gene FDX1 Predict the Prognosis and Correlate with Immune Infiltration in Clear Cell Renal Cell Carcinoma
Source: J Oncol. 2022 Dec 10;2022:2124088. doi: 10.1155/2022/2124088 (PMC9759391; doi:10.1155/2022/2124088)
Supplement: Supplementary Materials — Supplementary Figure 1: The correlation between DLAT, DLD, MTF1, LIAS, and OS in KIRC. Supplementary Figure 2: The correlation between DLAT, DLD, LIPT1, MTF1, LIAS, PDHA1, GLS, and PFS in KIRC. Supplementary Figure 3: Correlation between the expression of FDX1 and clinical features. Supplementary Figure 4: The exploration of underlying mechanism of low expression of FDX1 in tumor tissues. Supplementary Figure 5: Differential analysis between the FDX1 high expression group and the FDX1 low expression group. Supplementary Figure 6: Tumor mutational burden (TMB), immune infiltration, and drug susceptibility. [file 2124088.f1.zip › Supplementary Materials.docx]

**Supplementary Materials**

**Supplementary Figures**


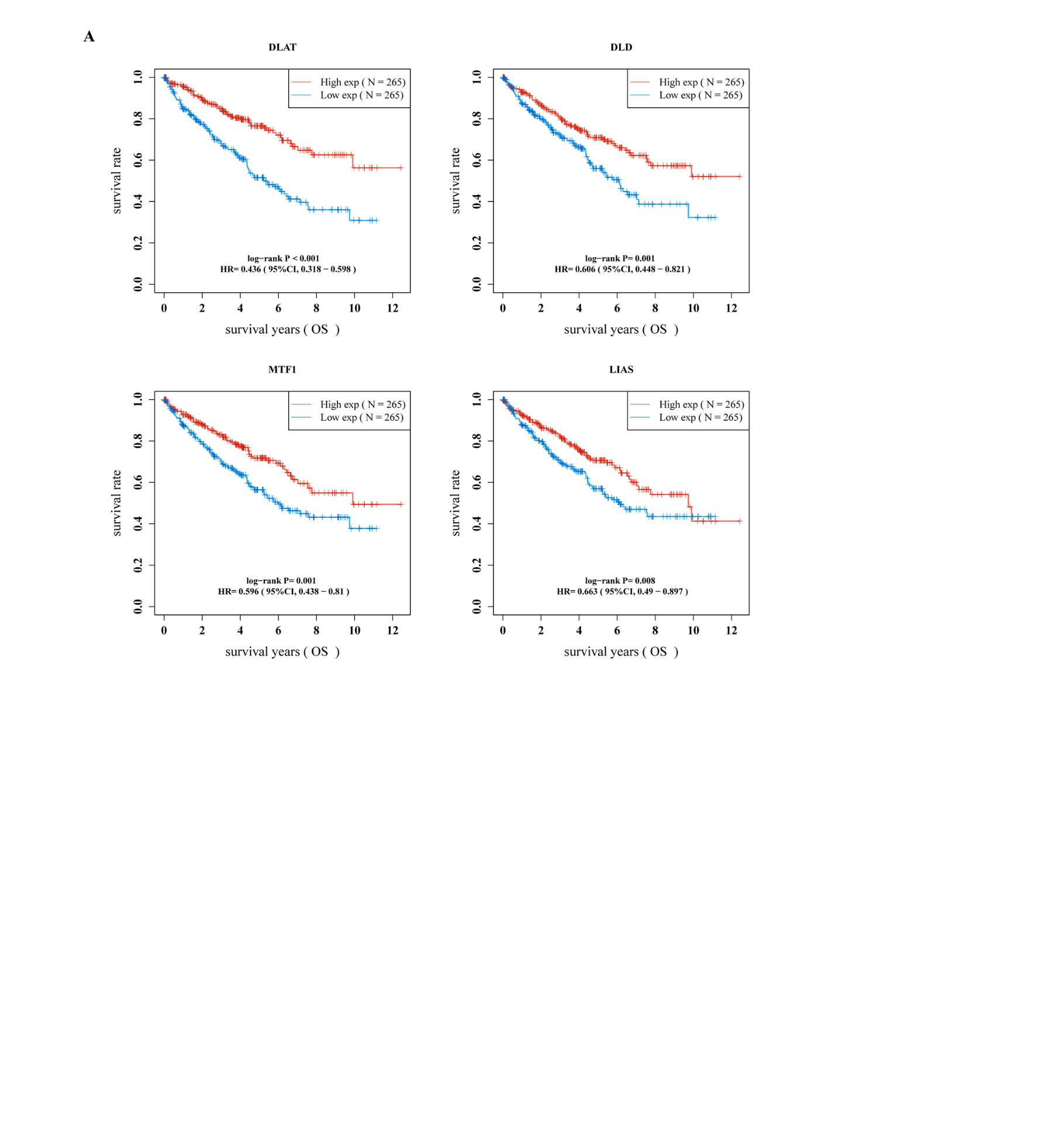


**Supplementary Figure 1** The correlation between *DLAT*, *DLD*, *MTF1*, *LIAS* and OS in KIRC. The curve comparison with the log-rank test revealed statistically significant differences as shown on graph. OS, overall survival. KIRC, kidney clear cell renal cell carcinoma.


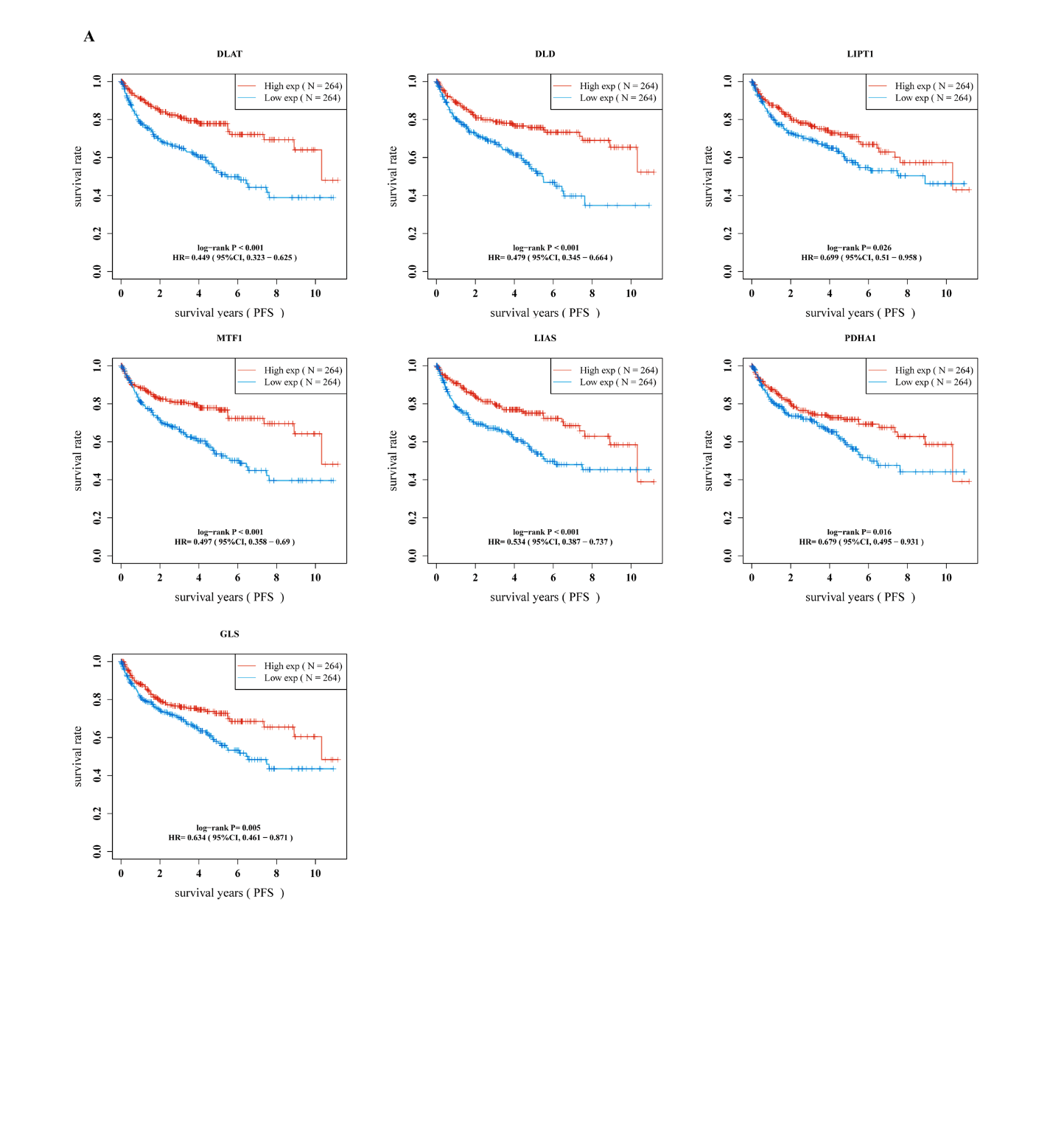


**Supplementary Figure 2** The correlation between *DLAT*, *DLD*, *LIPT1*, *MTF1*, *LIAS*, *PDHA1*, *GLS* and PFS in KIRC. The curve comparison with the log-rank test revealed statistically significant differences as shown on graph. PFS, progression free survival. KIRC, kidney clear cell renal cell carcinoma.


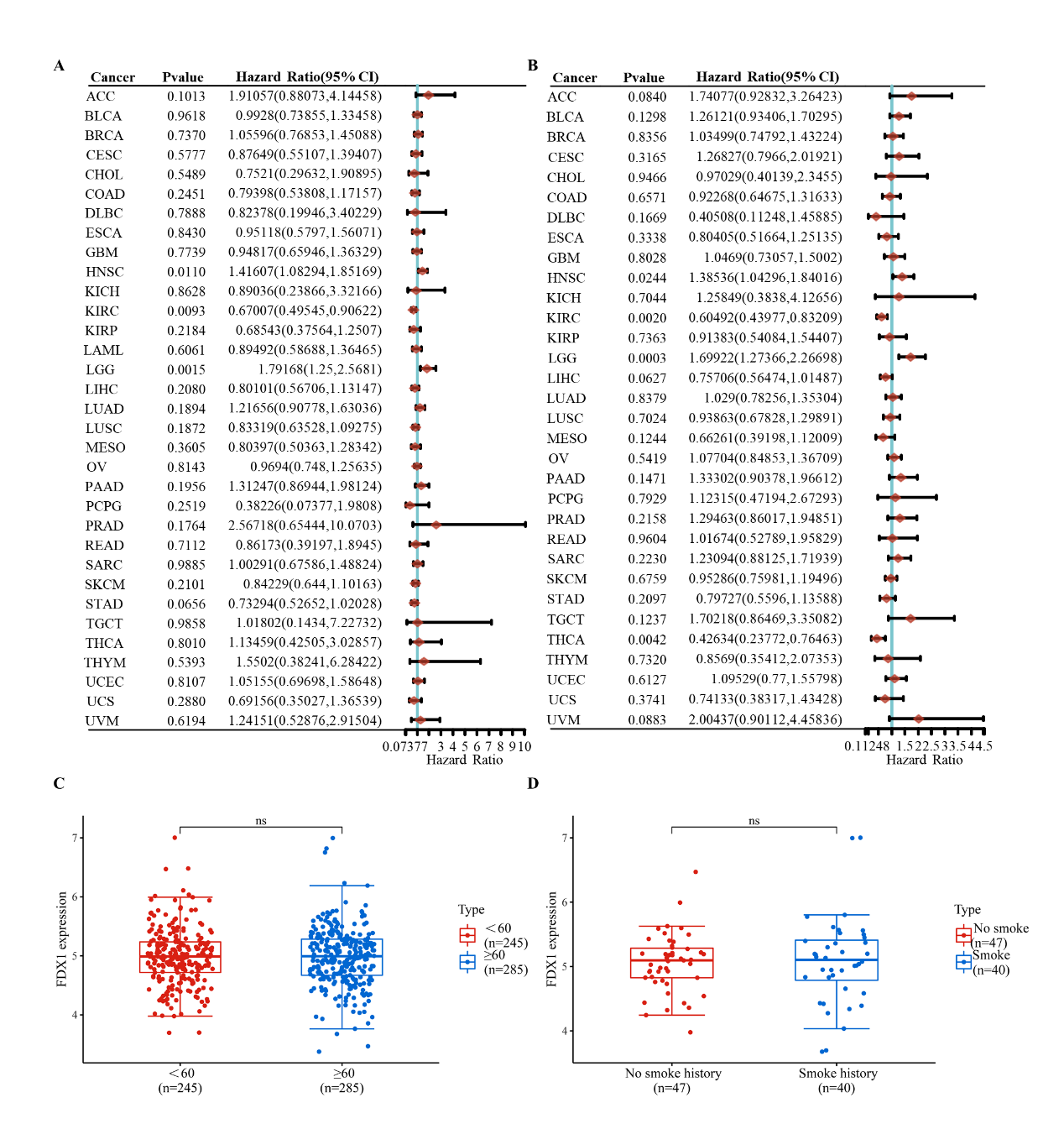


**Supplementary Figure 3** Correlation between the expression of *FDX1* and clinical features. **(A-B)** The pan-cancer analysis of OS and PFS in 33 cancer types between different *FDX1* expression groups. **(C-D)** Correlations of the expression of *FDX1* and age or smoking history. The statistical differences between different groups were compared through the Wilcox test. **p* < 0.05, ***p* < 0.01, ****p* < 0.001. OS, overall survival. PFS, progression free survival.


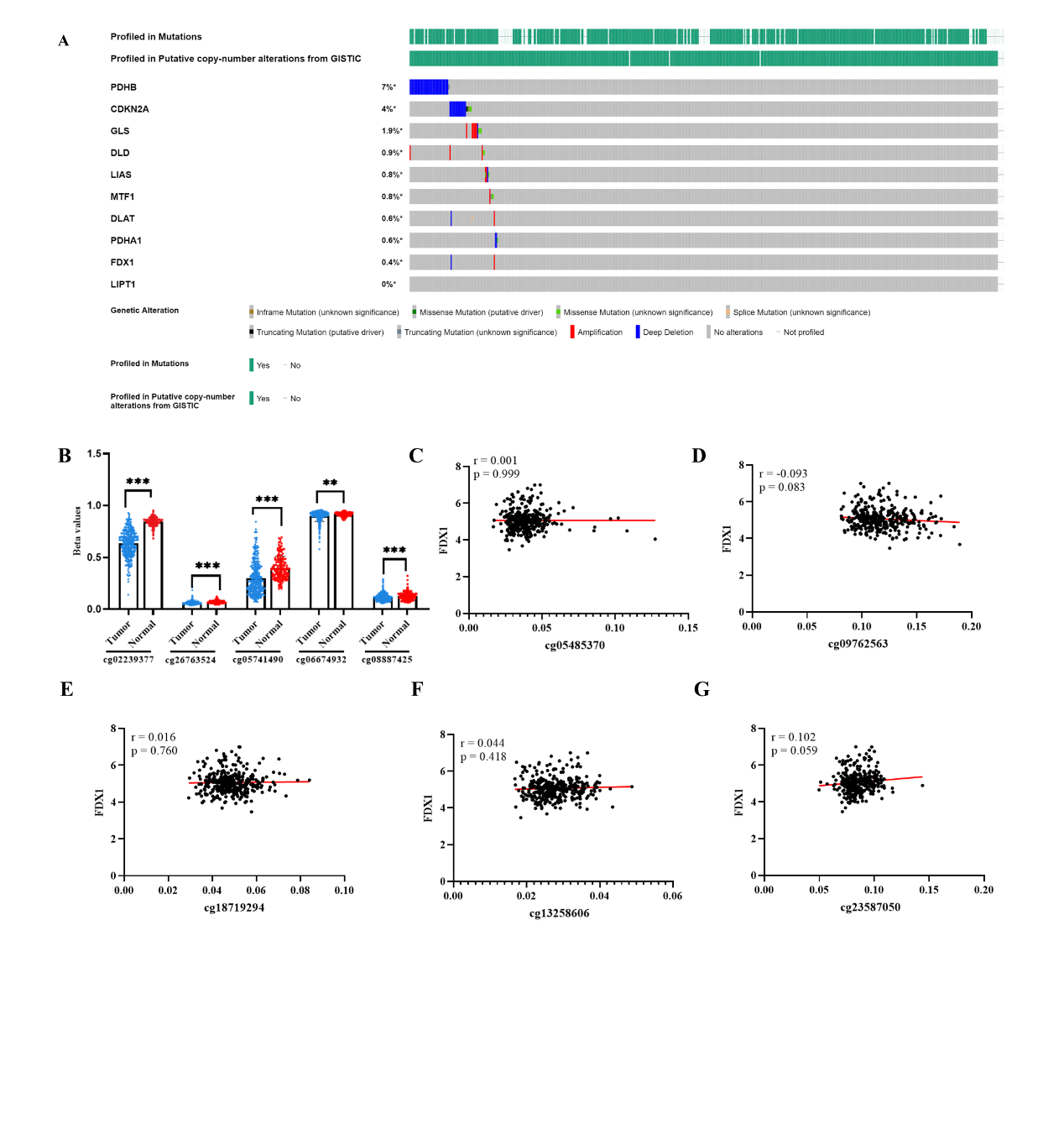


**Supplementary Figure 4** The exploration of underlying mechanism of low expression of *FDX1* in tumor tissues. **(A)** The mutation landscape of 10 CRGs in KIRC. **(B)** Statistical comparison of the difference in methylation levels of 6 hypomethylation CpG sites in tumor. The statistical differences between different groups were compared through the Wilcox test. **p* < 0.05, ***p* < 0.01, ****p* < 0.001. **(C-G)** Correlation between *FDX1* expression and cg05485370, cg09762563, cg18719294, cg13258606, cg2587050 methylation levels.


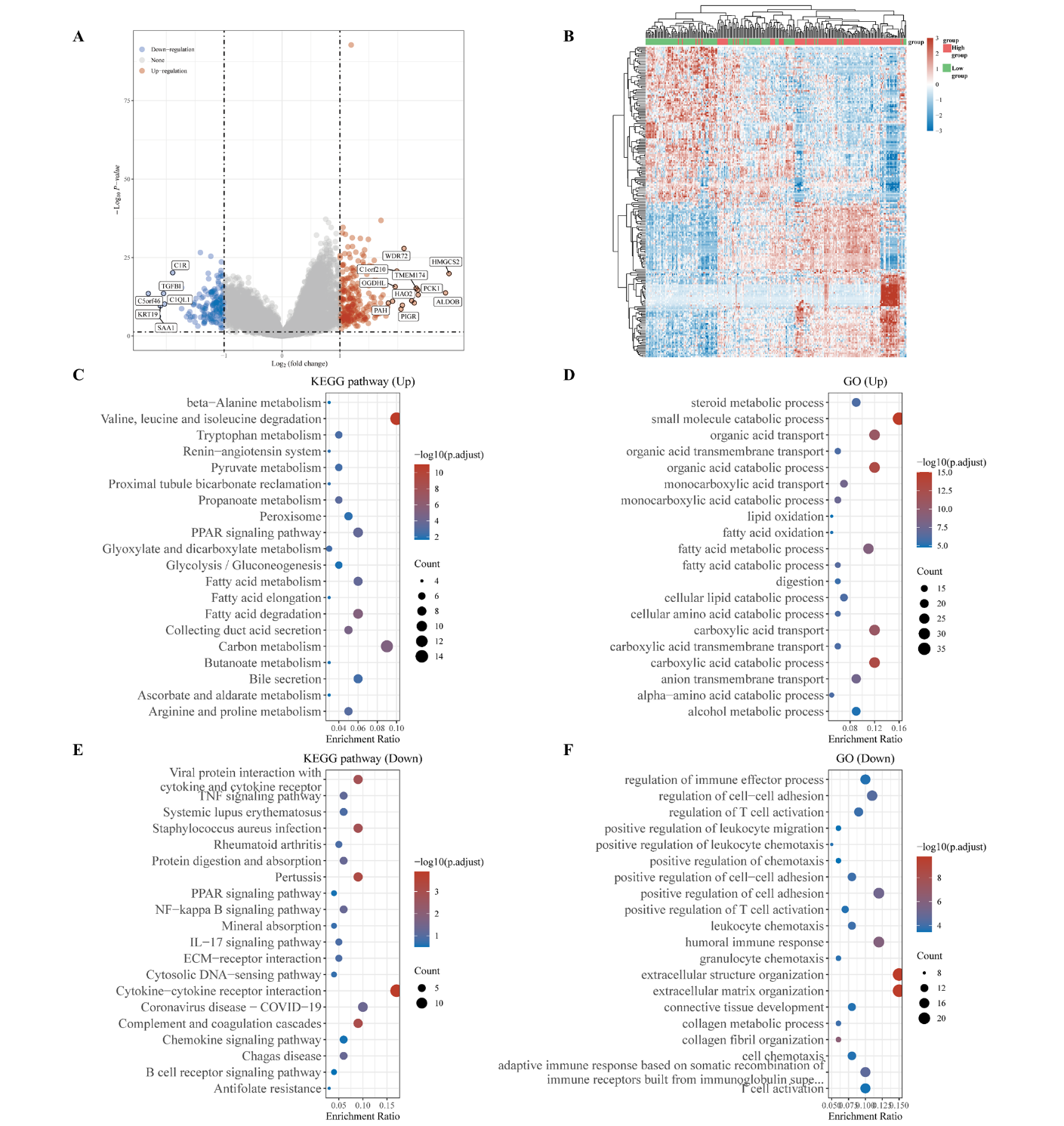


**Supplementary Figure 5** Differential analysis between *FDX1* high expression group and low expression group. **(A)** The volcano plot of the differential gene expression. Red plot, the upregulated genes in high expression group. Blue plot, the downregulated genes in low expression group. **(B)** The heatmap of the differential gene expression. The top 50 upregulated and downregulated genes were showed. **(C-F)** The KEGG and GO functional analysis of upregulated genes and downregulated genes. Colors represent the significance of differential enrichment.


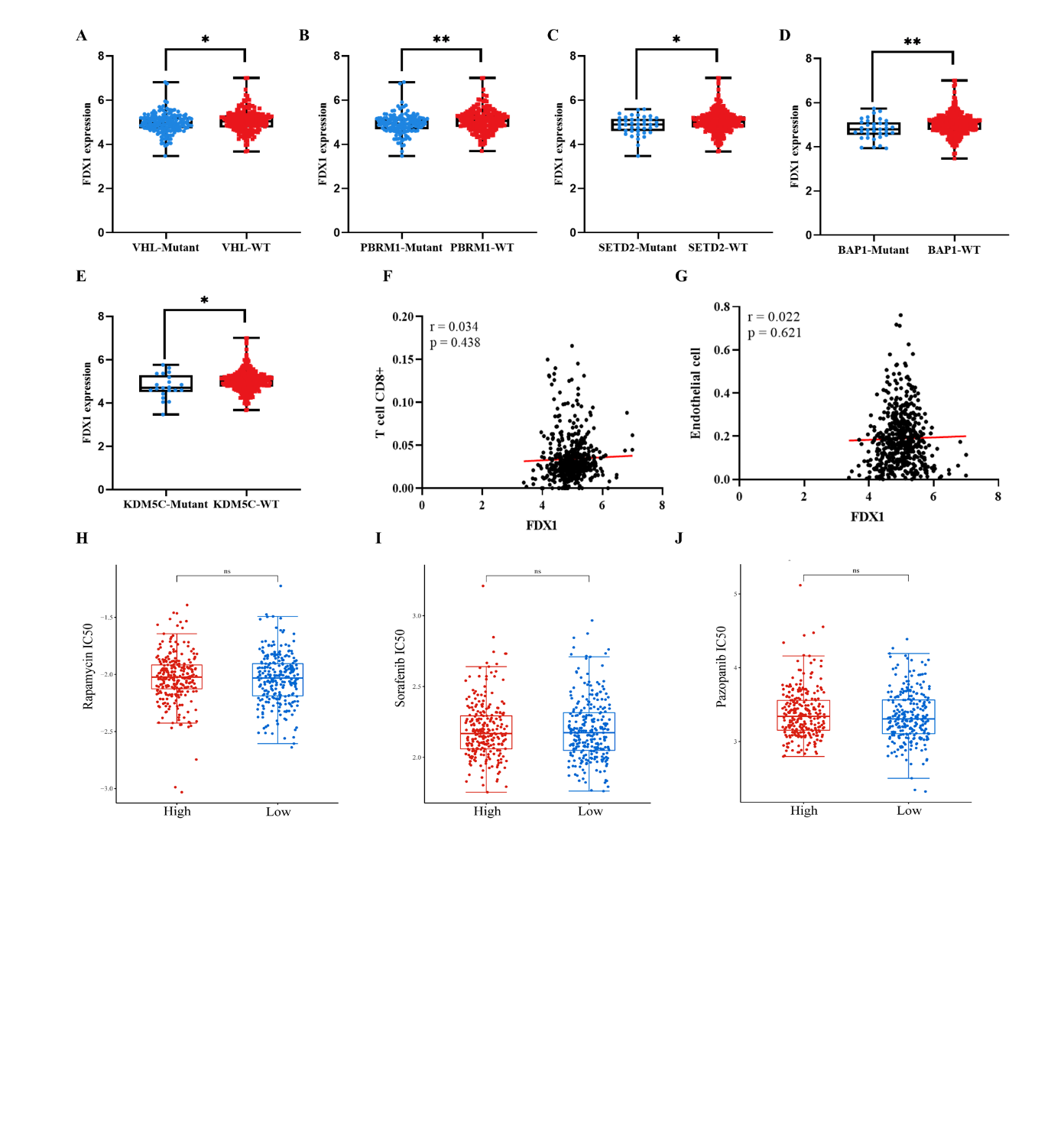


**Supplementary Figure 6** Tumor mutational burden (TMB), immune infiltration and drug susceptibility. **(A-E)** The expression of *FDX1* between *VHL*, *PBRM1*, *SETD2*, *BAP1*, *KDM5C* mutation groups and wild type groups. **(F-G)** The correlation between *FDX1* expression and infiltration levels of T cell CD8+, and endothelial cell. **(H-J)** Sensitivity analysis for rapamycin, sorafenib and pazopanib in *FDX1* high expression groups and low expression groups. The statistical differences between different groups were compared through the Wilcox test. **p* < 0.05, ***p* < 0.01, ****p* < 0.001. GO, Gene Ontology; KEGG, Kyoto Encyclopedia of Genes and Genomes.
