# Supplementary figures and images for: A Novel Cuproptosis-Related Prognostic Model and the Hub Gene FDX1 Predict the Prognosis and Correlate with Immune Infiltration in Clear Cell Renal Cell Carcinoma
Source: J Oncol. 2022 Dec 10;2022:2124088. doi: 10.1155/2022/2124088 (PMC9759391; doi:10.1155/2022/2124088)

A

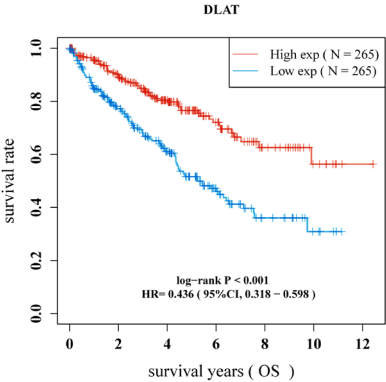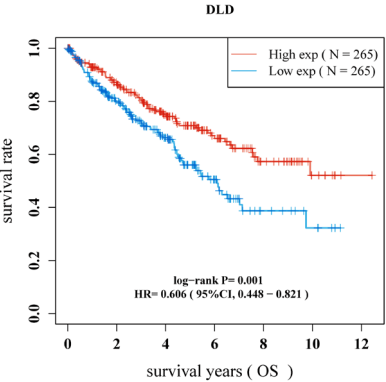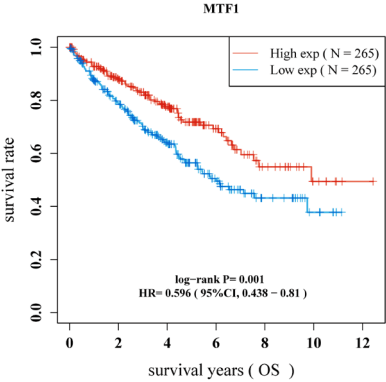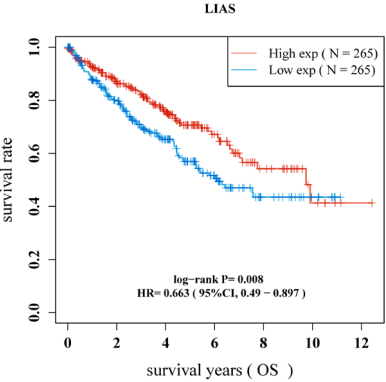

Supplement: Supplementary Materials — Supplementary Figure 1: The correlation between DLAT, DLD, MTF1, LIAS, and OS in KIRC. Supplementary Figure 2: The correlation between DLAT, DLD, LIPT1, MTF1, LIAS, PDHA1, GLS, and PFS in KIRC. Supplementary Figure 3: Correlation between the expression of FDX1 and clinical features. Supplementary Figure 4: The exploration of underlying mechanism of low expression of FDX1 in tumor tissues. Supplementary Figure 5: Differential analysis between the FDX1 high expression group and the FDX1 low expression group. Supplementary Figure 6: Tumor mutational burden (TMB), immune infiltration, and drug susceptibility. [file 2124088.f1.zip › Supplementary Figure 1.pdf]

A

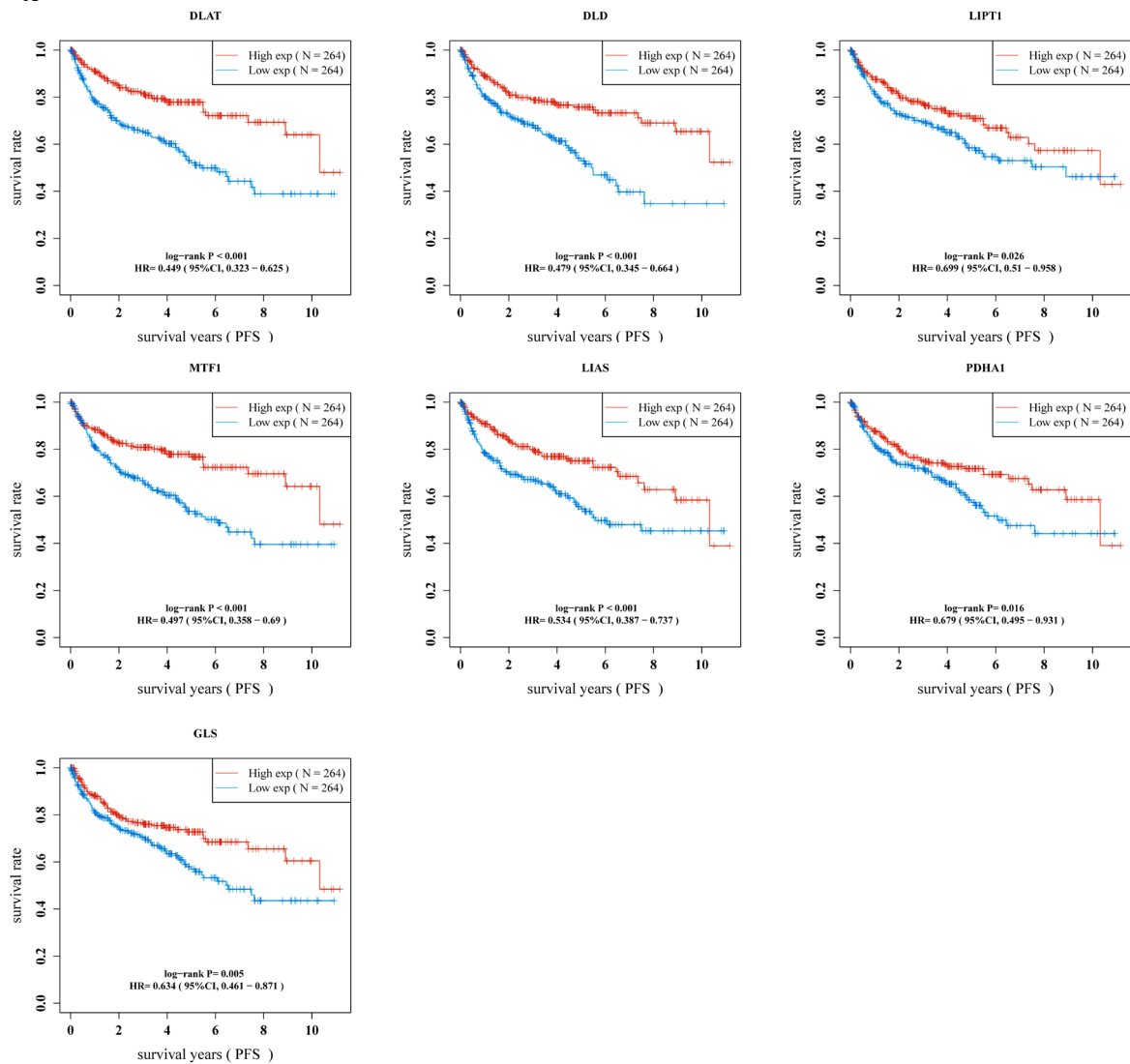

Supplement: Supplementary Materials — Supplementary Figure 1: The correlation between DLAT, DLD, MTF1, LIAS, and OS in KIRC. Supplementary Figure 2: The correlation between DLAT, DLD, LIPT1, MTF1, LIAS, PDHA1, GLS, and PFS in KIRC. Supplementary Figure 3: Correlation between the expression of FDX1 and clinical features. Supplementary Figure 4: The exploration of underlying mechanism of low expression of FDX1 in tumor tissues. Supplementary Figure 5: Differential analysis between the FDX1 high expression group and the FDX1 low expression group. Supplementary Figure 6: Tumor mutational burden (TMB), immune infiltration, and drug susceptibility. [file 2124088.f1.zip › Supplementary Figure 2.pdf]

A

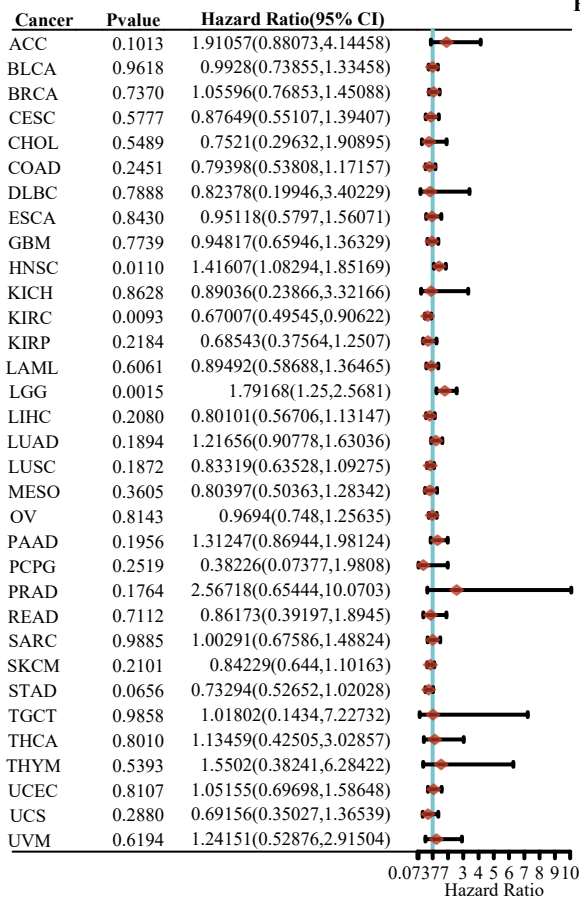

B

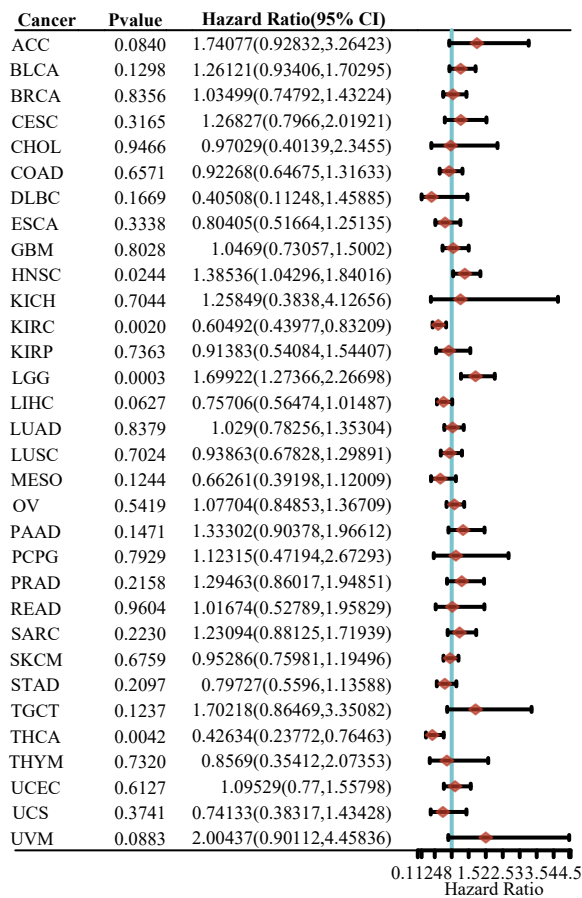

C

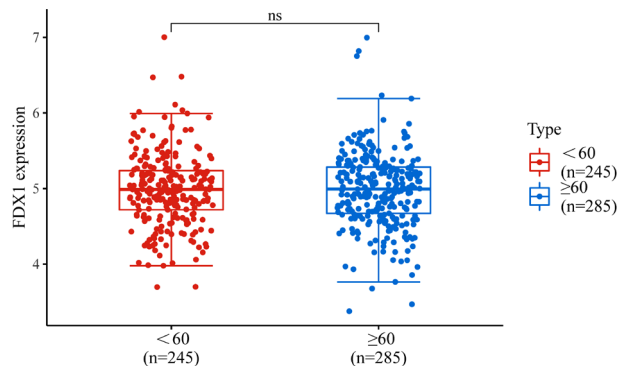

D

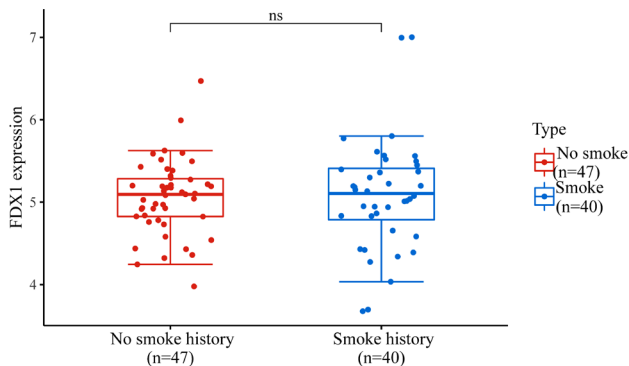

Supplement: Supplementary Materials — Supplementary Figure 1: The correlation between DLAT, DLD, MTF1, LIAS, and OS in KIRC. Supplementary Figure 2: The correlation between DLAT, DLD, LIPT1, MTF1, LIAS, PDHA1, GLS, and PFS in KIRC. Supplementary Figure 3: Correlation between the expression of FDX1 and clinical features. Supplementary Figure 4: The exploration of underlying mechanism of low expression of FDX1 in tumor tissues. Supplementary Figure 5: Differential analysis between the FDX1 high expression group and the FDX1 low expression group. Supplementary Figure 6: Tumor mutational burden (TMB), immune infiltration, and drug susceptibility. [file 2124088.f1.zip › Supplementary Figure 3.pdf]

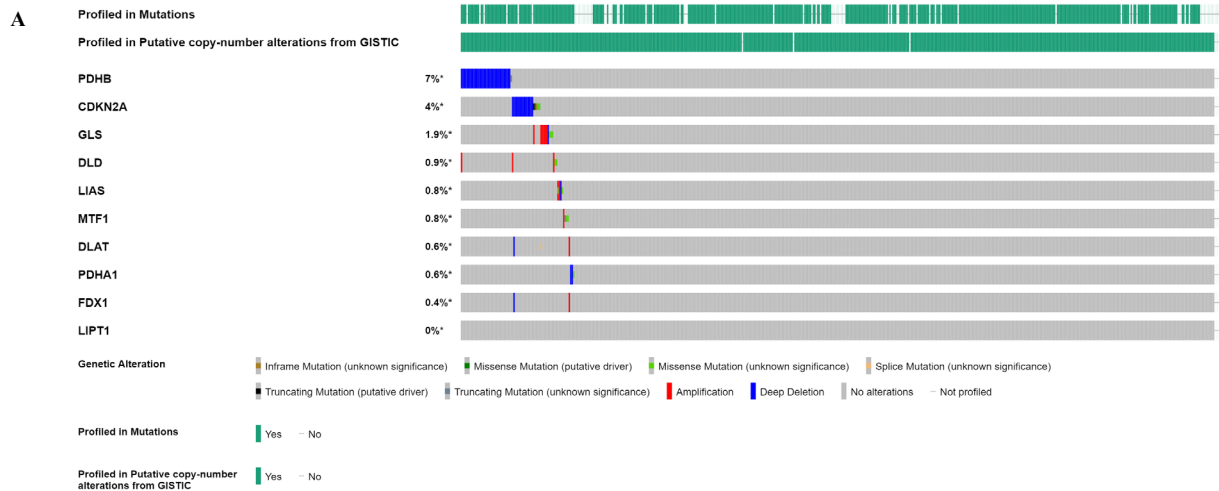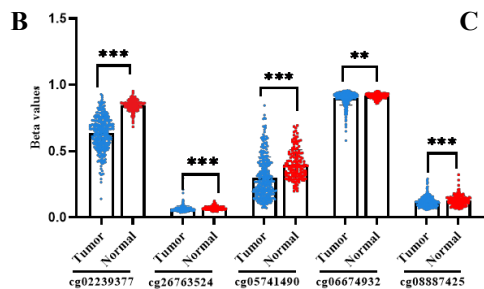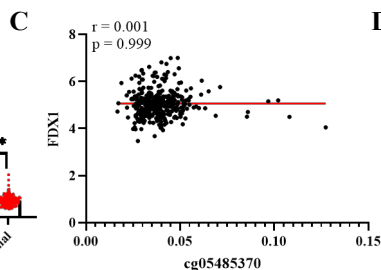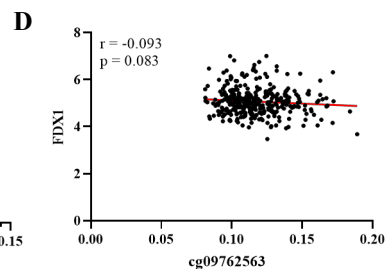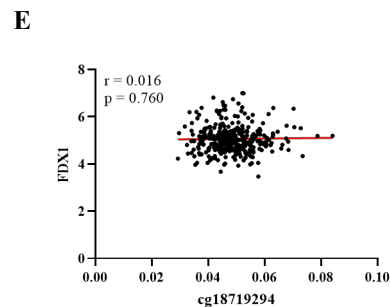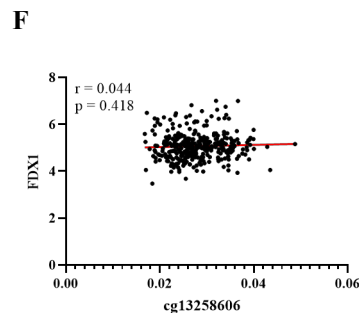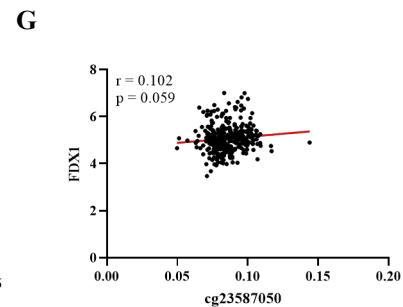

Supplement: Supplementary Materials — Supplementary Figure 1: The correlation between DLAT, DLD, MTF1, LIAS, and OS in KIRC. Supplementary Figure 2: The correlation between DLAT, DLD, LIPT1, MTF1, LIAS, PDHA1, GLS, and PFS in KIRC. Supplementary Figure 3: Correlation between the expression of FDX1 and clinical features. Supplementary Figure 4: The exploration of underlying mechanism of low expression of FDX1 in tumor tissues. Supplementary Figure 5: Differential analysis between the FDX1 high expression group and the FDX1 low expression group. Supplementary Figure 6: Tumor mutational burden (TMB), immune infiltration, and drug susceptibility. [file 2124088.f1.zip › Supplementary Figure 4.pdf]

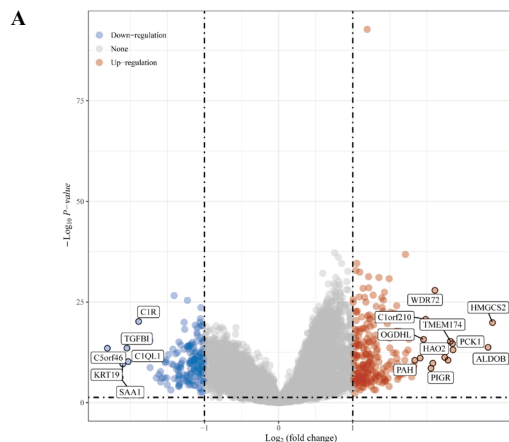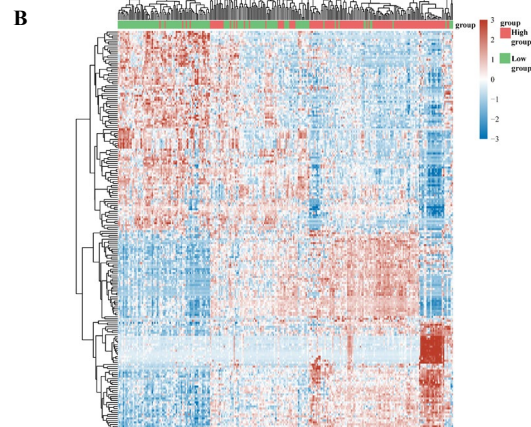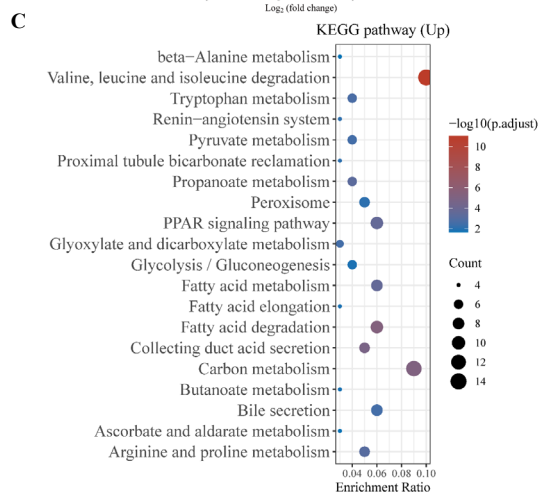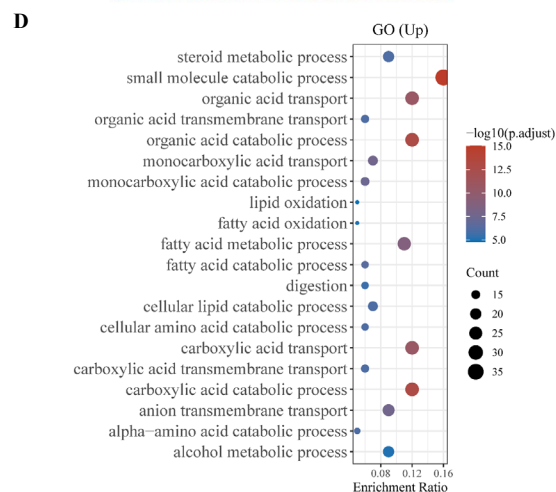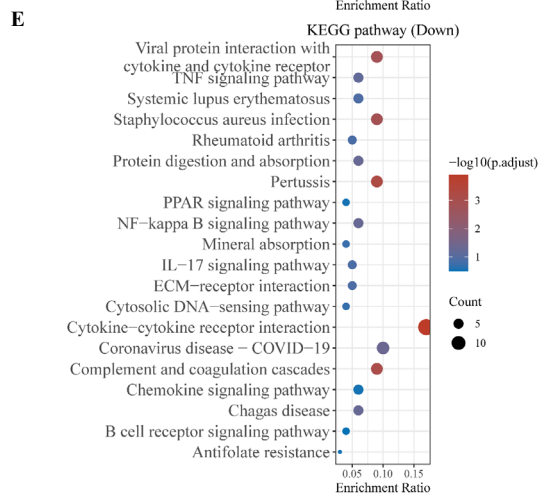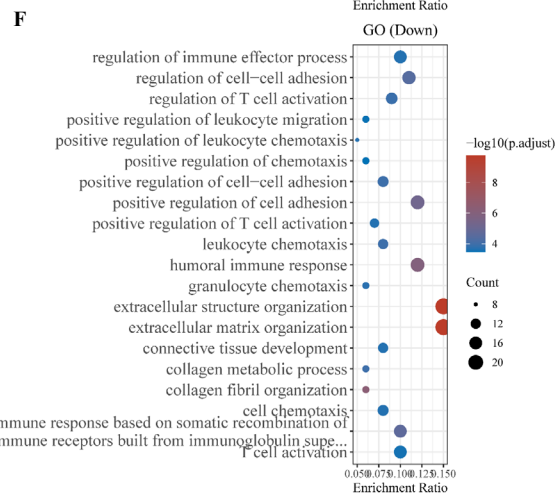

Supplement: Supplementary Materials — Supplementary Figure 1: The correlation between DLAT, DLD, MTF1, LIAS, and OS in KIRC. Supplementary Figure 2: The correlation between DLAT, DLD, LIPT1, MTF1, LIAS, PDHA1, GLS, and PFS in KIRC. Supplementary Figure 3: Correlation between the expression of FDX1 and clinical features. Supplementary Figure 4: The exploration of underlying mechanism of low expression of FDX1 in tumor tissues. Supplementary Figure 5: Differential analysis between the FDX1 high expression group and the FDX1 low expression group. Supplementary Figure 6: Tumor mutational burden (TMB), immune infiltration, and drug susceptibility. [file 2124088.f1.zip › Supplementary Figure 5.pdf]

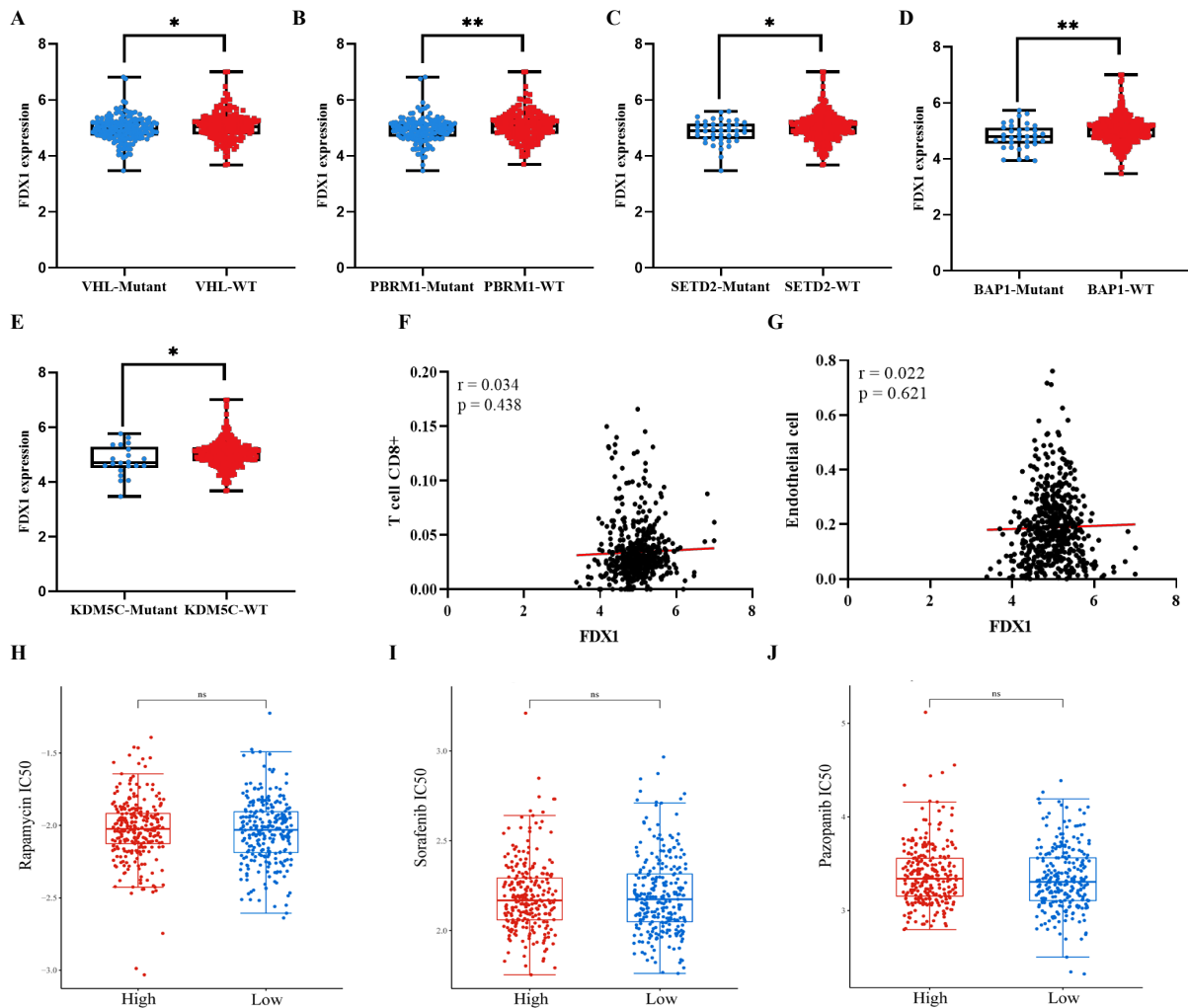

Supplement: Supplementary Materials — Supplementary Figure 1: The correlation between DLAT, DLD, MTF1, LIAS, and OS in KIRC. Supplementary Figure 2: The correlation between DLAT, DLD, LIPT1, MTF1, LIAS, PDHA1, GLS, and PFS in KIRC. Supplementary Figure 3: Correlation between the expression of FDX1 and clinical features. Supplementary Figure 4: The exploration of underlying mechanism of low expression of FDX1 in tumor tissues. Supplementary Figure 5: Differential analysis between the FDX1 high expression group and the FDX1 low expression group. Supplementary Figure 6: Tumor mutational burden (TMB), immune infiltration, and drug susceptibility. [file 2124088.f1.zip › Supplementary Figure 6.pdf]
